# Diagnostic implications of genetic copy number variation in epilepsy plus

**DOI:** 10.1111/epi.14683

**Published:** 2019-03-13

**Authors:** Antonietta Coppola, Elena Cellini, Hannah Stamberger, Elmo Saarentaus, Valentina Cetica, Dennis Lal, Tania Djémié, Magdalena Bartnik‐Glaska, Berten Ceulemans, J. Helen Cross, Tine Deconinck, Salvatore De Masi, Thomas Dorn, Renzo Guerrini, Dorotha Hoffman‐Zacharska, Frank Kooy, Lieven Lagae, Nicholas Lench, Johannes R. Lemke, Ersilia Lucenteforte, Francesca Madia, Heather C. Mefford, Deborah Morrogh, Peter Nuernberg, Aarno Palotie, An‐Sofie Schoonjans, Pasquale Striano, Elzbieta Szczepanik, Anna Tostevin, Joris R. Vermeesch, Hilde Van Esch, Wim Van Paesschen, Jonathan J Waters, Sarah Weckhuysen, Federico Zara, Peter De Jonghe, Sanjay M. Sisodiya, Carla Marini, EuroEPINOMICS‐RES Consortium, EpiCNV Consortium, Anna‐Elina Lehesjioki, Dana Craiu, Tiina Talvik, Hande Caglayan, Jose Serratosa, Katalin Sterbova, Rikke S. Møller, Helle Hjalgrim, Holger Lerche, Yvonne Weber, Ingo Helbig, Sarah von Spiczak, Carmen Barba, Anneleen Bogaerts, Antonella Boni, Elisabeth Caruana Galizia, Sara Chiari, Gianpiero Di Gacomo, Annarita Ferrari, Silvia Guarducci, Sabrina Giglio, Philip Holmgren, Costin Leu, Federico Melani, Francesca Novara, Marilena Pantaleo, Elke Peeters, Tiziana Pisano, Anna Rosati, Josemir Sander, Natasha Schoeler, Pawel Stankiewicz, Salvatore Striano, Arvid Suls, Monica Traverso, Geert Vandeweyer, Anke Van Dijck, Orsetta Zuffardi

**Affiliations:** ^1^ Department of Clinical and Experimental Epilepsy UCL Queen Square Institute of Neurology WC1N3BG UK; ^2^ The Chalfont Centre for Epilepsy Chesham Lane, Chalfont St Peter Bucks UK; ^3^ Epilepsy Centre Department of Neuroscience, Reproductive and Odontostomatological Sciences Federico II University Naples Italy; ^4^ Pediatric Neurology, Neurogenetics and Neurobiology Unit and Laboratories Neuroscience Department A Meyer Children‘s Hospital University of Florence Florence Italy; ^5^ Neurogenetics Group Center for Molecular Neurology VIB 2650 Antwerp Belgium; ^6^ Laboratory of Neurogenetics Institute Born‐Bunge University of Antwerp Antwerp Belgium; ^7^ Department of Neurology Antwerp University Hospital Antwerp Belgium; ^8^ Analytic and Translational Genetics Unit Massachusetts General Hospital Harvard Medical School Boston Massachusetts USA; ^9^ Stanley Center for Psychiatric Research Broad Institute of MIT and Harvard Cambridge Massachusetts USA; ^10^ Institute of Molecular Medicine Finland FIMM University of Helsinki Helsinki Finland; ^11^ Epilepsy Center Neurological Institute Cleveland Clinic Cleveland OH 44195 US; ^12^ Genomic Medicine Institute Lerner Research Institute Cleveland Clinic Cleveland OH 44195 US; ^13^ Cologne Center for Genomics University of Cologne Germany; ^14^ Department of Medical Genetics Institute of Mother and Child Warsaw Poland; ^15^ Department of Neurology‐Pediatric Neurology University and University Hospital Antwerp Antwerp Belgium; ^16^ Neurology Department Great Ormond Street Hospital NHS Foundation Trust London UK; ^17^ Clinical Neuroscience UCL GOSH Institute of Child Health London UK; ^18^ Young Epilepsy Lingfield UK; ^19^ Clinical Trial Office Meyer Children‘s Hospital Florence Italy; ^20^ Swiss Epilepsy Center Bleulerstrasse 60 CH‐8008 Switzerland; ^21^ Department of Medical Genetics University of Antwerp Antwerp Belgium; ^22^ Department of Development and Regeneration Section Pediatric Neurology University Hospital KU Leuven 3000 Leuven Belgium; ^23^ North East Thames Regional Genetics Service Great Ormond Street Hospital for Children NHS Foundation Trust London UK; ^24^ Institute of Human Genetics University of Leipzig Hospitals and Clinics Leipzig Germany; ^25^ Department of Clinical and Experimental Medicine University of Pisa, Italy Clinical Trial Office Meyer Children‘s Hospital Florence Italy; ^26^ Neurogenetic Laboratory Scientific Institute for Research, Hospitalisation and Health Care (IRCCS) G. Gaslini Institute Genova Italy; ^27^ Department of Pediatrics Division of Genetic Medicine University of Washington Seattle USA; ^28^ Pediatric Neurology and Muscular Diseases Unit DINOGMI‐Department of Neurosciences, Rehabilitation, Ophthalmology Genetics, Maternal and Child Health University of Genoa, ‘G. Gaslini’ Institute Genova Italy; ^29^ Clinic of Neurology of Children and Adolescents Institute of Mother and Child Warsaw Poland; ^30^ Center for Human Genetics University Hospitals Leuven Herestraat 49 3000 Leuven Belgium; ^31^ Department of Neurology University Hospitals Leuven Herestraat 49 3000 Leuven Belgium

**Keywords:** array CGH, copy number variants, epilepsy genes, SNP array

## Abstract

**Objective:**

Copy number variations (CNVs) represent a significant genetic risk for several neurodevelopmental disorders including epilepsy. As knowledge increases, reanalysis of existing data is essential. Reliable estimates of the contribution of CNVs to epilepsies from sizeable populations are not available.

**Methods:**

We assembled a cohort of 1255 patients with preexisting array comparative genomic hybridization or single nucleotide polymorphism array based CNV data. All patients had “epilepsy plus,” defined as epilepsy with comorbid features, including intellectual disability, psychiatric symptoms, and other neurological and nonneurological features. CNV classification was conducted using a systematic filtering workflow adapted to epilepsy.

**Results:**

Of 1097 patients remaining after genetic data quality control, 120 individuals (10.9%) carried at least one autosomal CNV classified as pathogenic; 19 individuals (1.7%) carried at least one autosomal CNV classified as possibly pathogenic. Eleven patients (1%) carried more than one (possibly) pathogenic CNV. We identified CNVs covering recently reported (*HNRNPU)* or emerging (*RORB*) epilepsy genes, and further delineated the phenotype associated with mutations of these genes. Additional novel epilepsy candidate genes emerge from our study. Comparing phenotypic features of pathogenic CNV carriers to those of noncarriers of pathogenic CNVs, we show that patients with nonneurological comorbidities, especially dysmorphism, were more likely to carry pathogenic CNVs (odds ratio = 4.09, confidence interval = 2.51‐6.68; *P* = 2.34 × 10^−9^). Meta‐analysis including data from published control groups showed that the presence or absence of epilepsy did not affect the detected frequency of CNVs.

**Significance:**

The use of a specifically adapted workflow enabled identification of pathogenic autosomal CNVs in 10.9% of patients with epilepsy plus, which rose to 12.7% when we also considered possibly pathogenic CNVs. Our data indicate that epilepsy with comorbid features should be considered an indication for patients to be selected for a diagnostic algorithm including CNV detection. Collaborative large‐scale CNV reanalysis leads to novel declaration of pathogenicity in unexplained cases and can promote discovery of promising candidate epilepsy genes.

1

**Key Points**

- CNV is an important contributor to the causation of epilepsy plus, with pathogenic and possibly pathogenic CNVs present in nearly 13% of cases
- The use of a specifically adapted workflow to classify CNVs allows the analysis of data from retrospectively collected patients screened through different platforms
- This study highlights CNVs covering recently reported (*HNRNPU*) or emerging (*RORB*) epilepsy genes, and further delineates the associated phenotype
- Patients with nonneurological comorbidities, especially dysmorphism, were more likely to carry pathogenic CNVs

## 2 INTRODUCTION

Current estimates suggest that genetics contribute to causation in 50%‐70% of the epilepsies.^1^ Copy number variations (CNVs) represent a prominent type of variant carrying risk for certain epilepsies.^2^, ^3^, ^4^, ^5^ Whole genome oligonucleotide array CGH or SNP array is routinely included in evaluation of patients with complex phenotypes with a suspected genetic cause.^6^ CNVs, as a risk factor or cause, have been reported in ~5%‐12% of patients with different types of epilepsies.^2^, ^4^, ^5^, ^7^, ^8^, ^9^ The risk of a pathogenic CNV is reportedly increased with concurrent intellectual disability (ID), dysmorphic features, autism spectrum disorder (ASD), drug resistance, or other comorbidities, from a study of 222 patients.^10^ Recurrent CNV “hotspots” predispose to different types of epilepsies.^5^, ^11^ CNV detection has pointed to novel epilepsy genes.^12^

Robust estimates of the frequencies and types of putatively relevant CNVs in epilepsy are needed to determine whether CNV detection should be included in genetic evaluation of patients with various epilepsy phenotypes. As knowledge of epilepsy genetics increases, systematic, iterative reevaluation of genetic data becomes essential. This process requires large numbers of individuals to be corralled, and because such data will inevitably come from different centers using different technologies, a robust means of joint reevaluation is essential.

Epilepsy is often a feature of neurodevelopmental disorders (NDDs). A recent study on individuals with NDDs and epilepsy reported similar results for rare variant frequency for individuals ascertained to have epileptic encephalopathy (EE) and for individuals ascertained for NDDs with unspecified epilepsy,^13^ suggesting that, genetically, epilepsy can be considered part of the spectrum of NDDs. Looking at this concept from the perspective of CNV, and to determine the frequency of CNVs in particular epilepsy phenotypes, we assembled a large international cohort of patients with the phenotype of “epilepsy plus,” which we define as the occurrence of epilepsy and comorbid features, including ID and psychiatric, neurological, and nonneurological features. Preexisting array data were systematically investigated using a workflow based on current knowledge of CNV classification. The workflow enabled combination of multicenter CNV data to provide a robust, up‐to‐date reevaluation of the contribution of CNVs to epilepsy plus and identified new candidate pathogenic autosomal CNVs. The method can be applied iteratively with additional cohorts at future time points, making optimal use of existing data.

## 3 MATERIALS AND METHODS

### 3.1 Ethics

This study was approved by the ethics committees of the participating centers. Written informed consent was provided by the patient, or the parent or the guardian of each patient as appropriate.

### 3.2 Data collection

Preexisting CNV data, derived from array CGH or SNP array conducted for clinical or research purposes, were collected from eight specialist epilepsy and/or genetic centers (Table S1). All patients also had comorbid features including ID, autism, dysmorphic features, other neurological or nonneurological conditions, structural brain abnormalities, or multidrug resistance.^14^ Clinical information was collected through referring clinicians. Seizure and epilepsy/syndrome types were classified according to the International League Against Epilepsy criteria when available.^15^

### 3.3 CNV analysis: Quality control and classification

**Figure 1:**
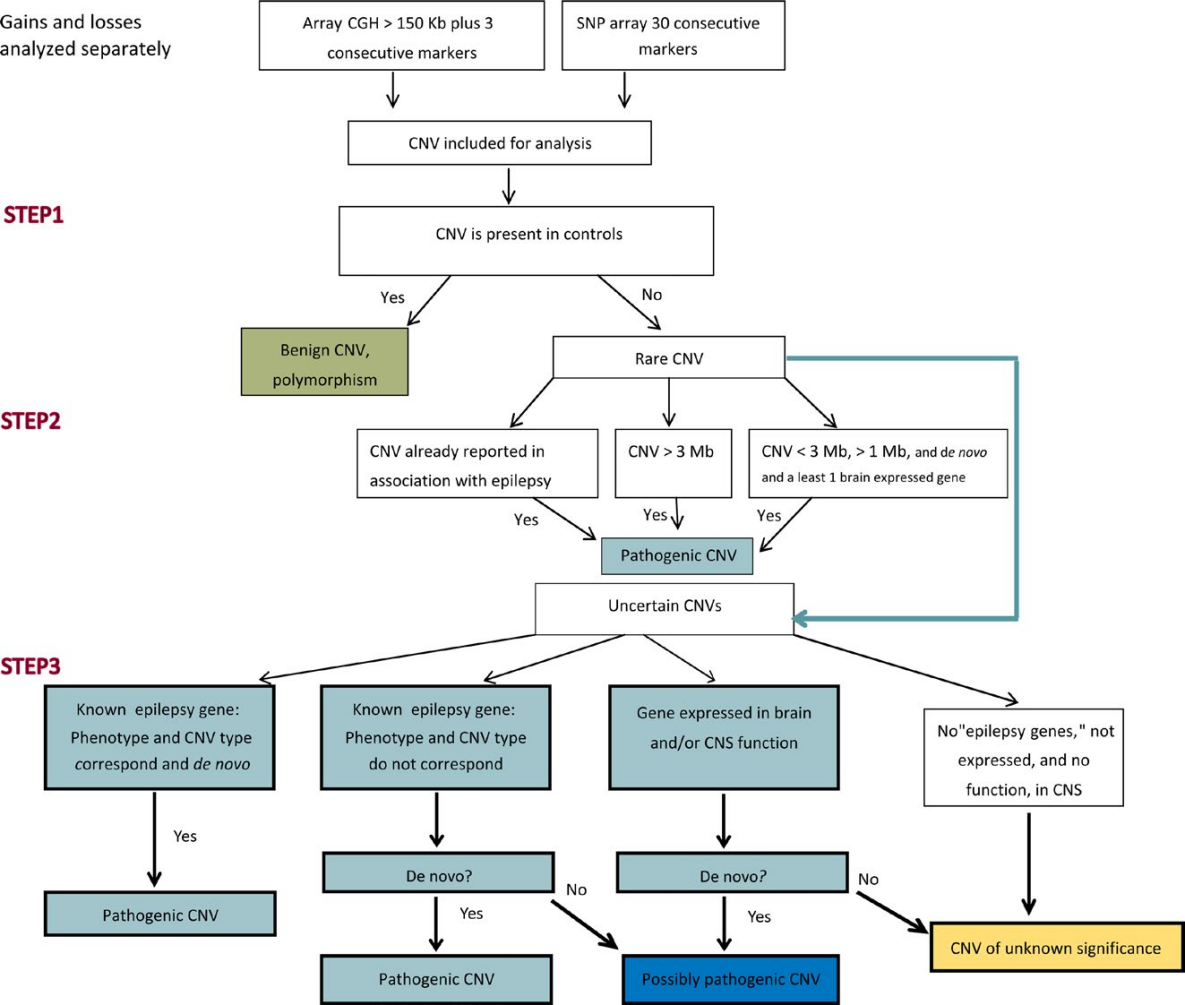
Workflow used to classify the copy number variations (CNVs) in our cohort of patients with epilepsy plus. Stepwise procedures are shown for CNV classification into benign, pathogenic, possibly pathogenic, and unknown significance groups. CGH, comparative genomic hybridization; CNS, central nervous system; SNP, single nucleotide polymorphism

All CNV calls were provided by the contributing centers (Table S1). Figure 1 shows the workflow we used to classify CNVs (Data S1). We focused only on autosomal CNVs due to higher quality of CNV calls from nonsex chromosomes.^16^ To ensure high reliability, we considered only CNVs with high calling confidence according to the following criteria: (1) size ≥ 150 kb, (2) coverage of ≥30 consecutive probes for SNP arrays and ≥3 probes for array CGH, and (3) microdeletion/microduplication frequency < 1% in the entire study sample. Samples with a total number of deletion or duplication (or both) calls >2 SD from the mean number of any calls/sample across the entire dataset were excluded from the analysis. Further manual analysis used a bespoke workflow based on current understanding of classification,^17^, ^18^ including the American College of Medical Genetics guidelines^19^ and additional literature.^2^, ^3^, ^4^, ^5^, ^6^, ^11^ CNVs were classified into four groups: pathogenic, possibly pathogenic, benign, or of unknown significance. Briefly, the workflow was as follows: First, common CNVs, present in the healthy population,^20^ were classified as “benign.” All remaining CNVs were then classified as “pathogenic” if they met the following criteria: ≥80% overlap of the study CNV with any CNVs known to be associated with epilepsy; or, CNV with a size ≥ 3 Mb; or, CNV with a size < 3 Mb and > 1 Mb, and with de novo occurrence. The remaining CNVs were further classified according to their gene content. A CNV was classified as pathogenic when it involved a gene known to be associated with epilepsy (Table S2), the phenotype was concordant with that in the literature, and the type of CNV (deletion/duplication) matched current knowledge on the pathogenic mechanism of the gene change (gain or loss of function). If a CNV contained a gene associated with epilepsy but the other conditions were not fulfilled, the CNV was considered pathogenic only if proven to be de novo and was otherwise classified as “possibly pathogenic.” CNVs containing a brain‐expressed gene, according to the published datasets^21^ and the database GTEx (http://www.gtexportal.org/home/), were classified as “possibly pathogenic” only if de novo. Analysis of recessive inheritance of epilepsy genes was not considered due to limitations of most CNV platforms on calling homozygous deletions or duplications. The remaining CNVs were classified as “of unknown significance.”

### 3.4 CNV confirmation

In each individual, the proposed possibly pathogenic CNVs were confirmed if DNA was available and inheritance status was confirmed using a second, locally developed technique for multiplex amplicon quantification (MAQ; Data S1; Agilent, https://www.agilent.com/en/products/next-generation-sequencing/amplicon-target-amplification-(multiplicom)/maq-overview).

### 3.5 Phenotype enrichment analysis

Using Fisher's exact test, we investigated whether patients carrying a pathogenic autosomal CNV, compared to those not carrying a pathogenic CNV, had overrepresentation for specific phenotype components (nonneurological disorders, neurological or psychiatric disorder, ID, facial dysmorphism, brain abnormalities, epilepsy onset < 1 year of age, and EE). The analysis was conducted in two ways—(1) for any pathogenic CNV and (2) for only large (>1 Mb) pathogenic CNVs—and was corrected for multiple testing accordingly.

### 3.6 Meta‐analysis

To determine the impact of epilepsy on the probability of identifying pathogenic CNVs, we used the following strategy. First, we split our cohort into two subgroups including patients with (1) epilepsy and ID including autistic features and (2) epilepsy and other psychiatric/neurological disorders. We gathered two “historical control groups” through a systematic review of the literature and a meta‐analysis and estimated the yield of pathogenic CNVs in patients with (1) ID including autistic features (without epilepsy) and (2) psychiatric/neurological disorders (without epilepsy). Then, we compared the yield of pathogenic CNVs between these groups with (1) epilepsy and ID including autistic features versus the historical‐control group with only ID and autistic features and (2) epilepsy with other psychiatric/neurological disorders versus the corresponding control group from the literature manifesting only other psychiatric/neurological disorders. We used the Cochran Q test to assess heterogeneity across studies.

To evaluate whether epileptic encephalopathies might specifically contribute to the yield of pathogenic CNVs, we compared patients with epilepsy manifesting as EE (epilepsy‐EE) from our cohort to those with epilepsy without EE (epilepsy‐notEE) from a systematic review of the literature.

The full search strategy, inclusion criteria, and methods are available in Data S1.

## 4 RESULTS

We assembled a cohort of 1255 patients. After quality control, 1097 patients were retained for analysis. Of these, 139 (12.7%) carried a total of 142 autosomal CNVs classified as either pathogenic (n = 122, 10.9%) or possibly pathogenic (n = 20, 1.7%). Eleven patients (1%) carried two pathogenic or possibly pathogenic CNVs (Table S3).

### 4.1 Pathogenic CNVs

To simplify presentation, we further divided pathogenic CNVs into four subgroups: (1) recurrent CNVs with well‐documented enrichment in epilepsy; (2) CNVs related to a genetic Online Mendelian Inheritance in Man database (OMIM) syndrome with neurological symptoms in which epilepsy can feature; (3) CNVs not known to be enriched in epilepsy and not associated with any other OMIM syndrome, but containing at least one gene that is already implicated in epilepsy; and (4) CNVs based on size combined with de novo occurrence.

#### 4.1.1 Recurrent CNVs with well‐documented enrichment in epilepsy

Thirty‐six individuals had a CNV known to be recurrent in people with epilepsy (36/120, 30%; Table 1).^2^, ^5^, ^11^ One individual had two recurrent pathogenic CNVs. The 16p13.11 deletion was the most frequent, occurring in 10 of 120 (8.3%) patients bearing pathogenic CNVs and 10 of 1097 (0.9%) of the studied individuals. Other frequently represented CNVs were 1p36 deletion (OMIM #607872, 5/120 patients, 4.2%), 15q11.2 deletion (OMIM #615656, 5/120 patients, 4.2%), and 22q11.2^19^ duplication (OMIM #608363, 5/120 patients, 4.2%).

**Table 1:** Recurrent CNVs with well‐documented enrichment in epilepsy

Samples, n	Chr region	CNV type	Syndrome	OMIM/reference boundaries	OMIM or references
5	1p36	Deletion	Chromosome 1p36 deletion syndrome	1:1‐27 600 000	#607872
2	1q21.1	Deletion	Chromosome 1q21.1 deletion syndrome	1:143 200 000‐147 500 000	#612474
1	1q21.1	Duplication	Chromosome 1q21.1 duplication syndrome	1:143 200 000‐147 500 000	#612475
5	15q11.2	Deletion	Chromosome 15q11.2 deletion syndrome	15:20 500 000‐25 500 000	#615656
3	15q13.3	Deletion	Chromosome 15q13.3 deletion syndrome	15:30 900 000‐33 400 000	#612001
3	16p11.2	Deletion	Chromosome 16p11.2 deletion syndrome	16:28 500 000‐35 300 000	#611913
10	16p13.11	Deletion	Chromosome 16p13.11 deletion syndrome	16:15 000 000‐16 300 000	Refs 3, 4
3	22q11.21	Deletion	Chromosome 22q11.2 deletion syndrome, distal	22:17 400 000‐25 500 000	#611867
5	22q11.21	Duplication	Chromosome 22q11.2 duplication syndrome	22:17 400 000‐25 500 000	#608363

CNV, copy number variation; OMIM, Online Mendelian Inheritance in Man database.

#### 4.1.2 CNVs related to a genetic OMIM syndrome with neurological symptoms in which epilepsy can feature

Thirty‐three individuals had pathogenic CNVs (33/120, 27.5%) mapping to regions for well‐characterized genetic syndromes associated with neurological features including epilepsy (Table S4a) and consistent with the relevant syndrome. The most frequent were as follows: the Williams‐Beuren 7q11.23 deletion syndrome (five patients), 15q11.2 duplication syndrome, distal (three patients), 16p11.2 duplication syndrome including *PRRT2* (four patients), the Potocki‐Lupski 17p11.2 duplication syndrome (two patients), and 17p13.3 deletion syndrome, also known as Miller‐Dieker lissencephaly deletion syndrome (three patients). We also identified de novo duplications at 2q24.3^22^ and at 4p16.3‐p13,^23^ for which regions both deletions and reciprocal duplications have been associated with epilepsy.^22^, ^23^

#### 4.1.3 CNVs including epilepsy‐related genes

Nineteen individuals had a CNV (19/120, 15.8%) including epilepsy‐related genes (Table 2). Five individuals had a CNV including *HNRNPU* (four de novo deletions and one duplication; two deletions and the duplication also contained the flanking *AKT3* gene). The four probands carrying deletions presented with epilepsy classified as Lennox‐Gastaut syndrome in one patient, genetic generalized epilepsy (GGE) in another, and early onset, drug‐resistant epilepsy not otherwise classified in the remaining two. Moderate to severe ID was reported in four patients and one also had ASD. Three had microcephaly, congenital and severe (−4 SD) in one. Brain magnetic resonance imaging showed corpus callosum agenesis or hypoplasia in three of four patients. Facial dysmorphic features were observed in three patients. The patient carrying a large (>100 Mb) duplication involving, among many other genes, *HNRNPU* and *AKT3*, had a complex phenotype including neonatal seizure onset, polymicrogyria, and multiple cardiac defects. Three individuals had a 9q21.13 deletion, one de novo and two of unknown inheritance, including a gene with recently described association with epilepsy, *RORB*. All the patients presented with ID and generalized epilepsy with absences or atypical absences, with eyelid myoclonia in two cases and photosensitivity in one. Further clinical details of patients with CNVs including *HNRNPU* or *RORB* are provided in Tables S5a and S5b. Three deletions encompassed the *ADGRV1* gene, two of which included *MEF2C*. Additional epilepsy genes that were found deleted or duplicated in single patients are listed in Table 2 and include *GNAO1*,* NEDD4L*, and *SIK1*.

**Table 2:** CNVs including epilepsy‐related genes

Individual	CNV type	Chr region	Start	Stop	Size, Mb	Inheritance	Epilepsy genes	Epilepsy phenotype	Other clinical features	Neuroimaging	Reported epilepsy phenotype associated with genes	Proposed disease mechanism (gain or loss of function) of reported epilepsy genes
IT_FLO_041	Deletion	1q42‐q44	236852056	249212809	12.4	De novo	*HNRNPU, AKT3*	Epilepsy NOS, DR	ID, stereotypies, congenital microcephaly (−4 SD), facial dysmorphism	CC agenesis, holoprosencephaly	*HNRNPU*: epileptic encephalopathy, early infantile, 54 (MIM 617391) *AKT*3: megalencephaly, polymicrogyria, polydactyly, hydrocephalus syndrome 2 (MIM 615937)	Loss of function (Table S6a); loss and Gain of function (Table S6a for more details)
BE_LEU_127	Duplication	1q21.1‐q44	144967252	249212666	104.2	Unknown	*HNRNPU, AKT3*	Epilepsy NOS with infantile onset, DR	Hypotonia, respiratory insufficiency, cardiac defects (large aorta ascendens and aortic arch, open ductus Botalli, ASD2 with small left/right shunt; pulmonary hypoplasia), kidney malrotation, facial dysmorphism	Widening of lateral ventricles and cavum vergae, polymicrogyria		
PO_W_031	Deletion	1q43‐q44	241757184	245072885	3.3	De novo	*HNRNPU, AKT3*	Focal of unknown origin	ID, hypotonia, acquired microcephaly (−2 SD), facial dysmorphism, hypotonia	Frontal lobe atrophy and CC hypoplasia		
IT_FLO_062	Deletion	1q44	244515959	247118959	2.6	De novo	*HNRNPU*	Generalized epilepsy, DR	ID, facial dysmorphism, GH deficit, deafness, acquired microcephaly (−2 SD), joint hyperlaxity, scoliosis	CC hypoplasia, ventricle asymmetry		
BE_LEU_009	Deletion	1q44	244823848	248093878	3.3	De novo	*HNRNPU*	Lennox‐Gastaut syndrome	ID, scoliosis, gastroesophageal reflux, bilateral corneal opacity	Delayed myelination, atrophic septum pellucidum, aqueduct stenosis, hydrocephaly		
BE_ANT_005	Deletion	2q24.3	163860225	172528095	8.7	De novo	*SCN1A, SCN2A*	Generalized epilepsy of unknown origin	ID, facial dysmorphism	Negative	*SCN1A*: epileptic encephalopathy, early infantile, 6 (Dravet syndrome; MIM 607208); epilepsy, generalized, with febrile seizures plus, type 2 (MIM 604403); febrile seizures, familial, 3A (MIM 604403); *SCN2A*: epileptic encephalopathy, early infantile, 11 (MIM 61372); seizures, benign familial infantile, 3 (MIM 6077451)	Loss of function; loss of function is associated with ASD, gain of function is associated with EE
IT_FLO_020	Deletion	5q14.3	88232244	90181244	1.9	De novo	*ADGRV1*	Epilepsy and FS NOS	None	Abnormal NOS	*ADGRV1*: febrile seizures, familial, 4 (MIM 604352); myoclonic epilepsy (Table S2); *MEF2C*: mental retardation, stereotypic movements, epilepsy, and/or cerebral malformations (MIM 613443)	Loss of function
PO_W_027	Deletion	5q14.3‐q15	87100153	92514871	5.4	De novo	*ADGRV1, MEF2C*	Epilepsy NOS	ID, dysmorphism	NA		
IT_FLO_024	Deletion	5q14q21	87770000	95780000	8	Unknown	*ADGRV1, MEF2C*	Epilepsy NOS	ID, macrocephaly, facial dysmorphism	Periventricular nodular heterotopia		
BE_LEU_211	Deletion	5q34	161059999	161446505	0.4	Unknown	*GABRA1, GABRA6*	Epilepsy NOS	ID	Corticosubcortical atrophy, supratentorial ventricular enlargement, periventricular vascular leukoencephalopathy, white matter lesions, lacunar infarcts in the basal ganglia and left thalamus	*GABRA1*: epileptic encephalopathy, early infantile, 19 (MIM 615744); possible susceptibility allele; juvenile myoclonic epilepsy (MIM 611136) and childhood absence epilepsy (MIM 611136); *GABRA6*: possible susceptibility allele for childhood absence epilepsy (Table S2)	Loss of function
PO_W_019	Deletion	9q21.13	74741400	77306932	2.6	De novo	*RORB*	Generalized photosensitive epilepsy (Jeavons syndrome)	ID, autism, strabismus	Negative	Generalized epilepsy, ID (Table S6b for more details)	Loss of function
BE_LEU_244	Deletion	9q21.13	76474486	81651005	5.2	Unknown	*RORB*	Generalized of unknown origin	ID, episodic ataxia	Small nonspecific white matter lesions over right parietal hemisphere		
US_267	Deletion	9q21.12‐q21.13	72702925	77128468	4.4	Unknown	*RORB*	Generalized epilepsy of unknown origin	ID, pyramidal sign, tremor, neurogenic bladder, psychotic episodes, severe macrocytic anemia, cold agglutinin disease, bilateral femuropatellar arthrosis, facial dysmorphisms	NA		
BE_LEU_205	Deletion	12p13.31	8691730	14215925	5.5	Unknown	*GRIN2B*	Focal epilepsy of unknown origin	ID, facial dysmorphism	Negative	Epileptic encephalopathy, early infantile, 27 (MIM 616139)	Loss and gain of function
PO_W_017	Duplication	14q11.2‐q12	23309096	31675172	8.3	De novo	*FOXG1*	Epilepsy NOS	ID	NA	Rett syndrome, congenital variant (MIM 613454)	Loss of function
IT_FLO_033	Deletion	16q12.1‐q21	52347499	64578499	12.2	Unknown	*GNAO1, GPR56*	Generalized epilepsy of structural origin	ID, language delay, facial dysmorphism, microcephaly, cryptorchidism	Polymicrogyria	*GNAO1*: epileptic encephalopathy, early infantile, 17 (MIM 615473); neurodevelopmental disorder with involuntary movements (MIM 617493); movement disorder with or without EE; *GPR56*: polymicrogyria (MIM 606854, 615752)	Loss of function; gain of function (recessive, loss of function)
IT_FLO_017	Deletion	18q21.31‐q21.33	54687002	59222020	4.5	Unknown	*NEDD4L*	Focal epilepsy of unknown origin	ID, hypotonia, dyspraxia, clumsiness, convergent strabismus	Vermis hypoplasia	OMIM: periventricular nodular heterotopia (MIM 617201; Lennox‐Gastaut syndrome–infantile spasms)	Loss of function
IT_FLO_074	Deletion	20q13.33	61845191	62893189	1.1	De novo	*KCNQ2, CHRNA4*	Generalized epilepsy of structural origin	Bilateral deafness, facial dysmorphism, lumbar kyphosis, sacral dimple, bilateral clinodactyly, small hands and fingers, hypoplastic flexion creases, atrial and ventricular septal defects, left renal agenesis defects, left renal agenesis	Periventricular nodular heterotopia	*KCNQ2*: epileptic encephalopathy, early infantile, 7 (MIM 613720); myokymia (MIM 121200); seizures, benign neonatal, 1 (MIM 121200); *CHRNA4*: epilepsy, nocturnal frontal lobe, 1 (MIM 600513)	Gain and loss of function; loss and gain of function
US_073	Deletion	21q22.3	43420839	46944323	3.5	Unknown	*SIK1*	Generalized epilepsy of unknown origin	ID, ataxia, spasticity, kyphoscoliosis, aortic valve deficiency	Enlarged lateral ventricles with pronunciation of occipital horns (colpocephaly)	Epileptic encephalopathy, early infantile, 30 (MIM 616341)	Loss of function

The reported phenotype associated with each known epilepsy gene refers to the phenotype reported in the OMIM or, if not available, the citation indicated in the supplementary material (Table S2).

ASD, atrial septal defects; CC, corpus callosum; CNV, copy number variation; DR, drug‐resistant; EE, epileptic encephalopathy; FS, febrile seizures; GH, growth hormone; ID, intellectual disability; MIM, Mendelian Inheritance in Man; NA, not available; NOS, not otherwise specified; OMIM, Online Mendelian Inheritance in Man database.

#### 4.1.4 Pathogenic autosomal CNVs based on size combined with de novo occurrence

Thirty‐two individuals (32/120, 26.6%) had CNVs that fell only into this category (one individual had two large pathogenic CNVs). We did not find overlapping CNVs in healthy individuals (Database of Genomic Variants; http://dgv.tcag.ca/dgv/app/home). Sixteen (16/32, 50%) of the CNVs ≥ 3 Mb showed an overlap or partial overlap with CNVs described in Decipher (https://decipher.sanger.ac.uk/) in patients exhibiting various clinical features including ID, seizures, and dysmorphisms (Table S4b). Interestingly, in a patient with an EE, we uncovered a de novo 13q33.1‐q13.3 deletion including *NBEA*.

#### 4.1.5 Possibly pathogenic CNVs

Nineteen individuals (19/1097, 1.73%) had a total of 20 CNVs classified as possibly pathogenic (one individual had two possibly pathogenic CNVs); 10 were de novo (Table 3). For 17 of 19 individuals (18/20 CNVs), DNA was available to check the CNV and/or inheritance using MAQ analysis. Eleven of the 18 analyzed CNVs were confirmed; in seven cases, the test was inconclusive (Table 3). These CNVs were classified as possibly pathogenic because they included an epilepsy gene but were inherited or the direction of the change was not concordant with the known disease mechanism (loss or gain of function) or phenotype, or because they included a brain‐expressed gene and were de novo. CNVs falling in the first category were a maternally inherited 10q23 deletion including *LGI1*, and a maternally inherited 20q13 duplication including *KCNQ2*,* CHRNA4*, and *EEF1A2*. Four other inherited CNVs included recessive genes: *PLCB1*,* TBC1D24*,* ABAT*, and *CNTNAP2*. A possible additional single nucleotide variant (SNV) on the other allele cannot be excluded. Of note, the *PLCB1* deletion was confirmed to be homozygous and would be considered pathogenic, but our flowchart was not developed for recessive analysis.

**Table 3:** Autosomal CNVs classified as “possibly pathogenic”

Individual	CNV type	Chr region	Start	Stop	Size, Mb	Inheritance	Proposed candidate genes^a^	Epilepsy phenotype	Other clinical features	Neuroimaging	MAQ validation
BE_LEU_009	Duplication	1q43	239842929	240356854	0.5	De novo	*FMN2* (start BP within gene), *CHRM3* (stop BP within gene)	Epilepsy NOS	ID, scoliosis, gastroesophageal reflux, bilateral corneal opacity	Delayed myelination, atrophic septum pellucidum, aqueduct stenosis, hydrocephaly	De novo
US_184	Duplication	3q28	191886383	192432844	0.5	De novo	*FGF12* (intragenic duplication)	Epilepsy NOS	Learning disabilities, attention deficit	Malrotation anterior and central part left hippocampus	NA
BE_LEU_141	Deletion	3q22.3	136035522	136412948	0.4	De novo	*STAG1, PCCB* (start BP within gene)	Epilepsy NOS	ID, autism, hypertonia, scoliosis,	NA	Confirmed in patient, absent in mother
IT_FLO_036^b^	Duplication	4q21.22‐q21.23	84035965	84813544	0.8	De novo	*COQ2* (stop BP within gene)	Myoclonic‐atonic epilepsy	ID	Negative	De novo
IT_FLO_127	Deletion	5q23.2	122481284	122987185	0.5	De novo	*CEP120, CSNK1G3* (stop BP within gene)	Myoclonic epilepsy	ID, hypotonia	Negative	De novo
PO_W_039	Duplication	7q35‐q36.1	146934489	148471787	1.5	Inherited (M)	*CNTNAP2* (start BPs within gene)	Epilepsy NOS	ID	CC hypoplasia	Inconclusive
IT_FL0_131	Deletion	6q26	161725639	161878527	0.2	De novo	*PARK2* (stop BP within gene)	Epilepsy NOS, FS	ID, hypotonia, obesity crowding of the fingers in both hands and feet, onychodystrophy	NA	Inconclusive
	Deletion	12p12.3	15469971	16375910	0.9	De novo	*STRAP*				
IT_FLO_109	Duplication	8p23.3‐p23.2	161272	801514	0.6	Unbalanced segregation of a balanced translocation (M)	*FBXO25*	Generalized epilepsy of unknown origin, DR	Language disorder	Negative	Inconclusive
IT_FLO_134	Deletion	8p23.3‐23.2	221611	801373	0.6	Unbalanced segregation of a balanced translocation (M)	*FBXO25*	Myoclonic epilepsy, FS	No	NA	Inconclusive
BE_LEU_236	Duplication	9q22.31	95208377	95590171	0.4	De novo	*BICD2*	Epilepsy NOS	ID	NA	De novo
IT_FLO_144	Deletion	10q23.33	95490322	95791986	0.3	Inherited (M)	*LGI1*	Epileptic encephalopathy NOS	Severe ID, quadriplegia, congenital cardiomyopathy (implanted pacemaker)	Cerebral atrophy microcephaly	Maternally inherited
BE_LEU_012	Duplication	15q13.2		32509932	1.6	Inherited (P)	*CHRNA7*	Focal of unknown origin	ID	No	Inconclusive
UK_L_056	Duplication	16p13.3	2481289	2888632	0.4	Unknown	*TBC1D24*	Myoclonic‐atonic epilepsy	ID	Negative	Confirmed in proband, parents NA
US_175	Deletion	16p13.2	8368145	8860296	0.5	Inherited (M)	*ABAT* (stop BP within gene)	Epileptic encephalopathy NOS	ID, apraxia, dyskinesia, generalized hypotonia	Negative	Maternally inherited
IT_FLO_023	Deletion	17q21.31	43160474	43922220	0.8	De novo	*NMT1* (start BP within gene), *PLCD3* (start BP within gene)	Focal epilepsy of unknown origin, FS	ID, macrocephaly, facial dysmorphism, cardiac defect, skin dyschromia	CC hypoplasia	Inconclusive
IT_FLO_083	Deletion	18q12.3	42605437	42784321	0.2	De novo	*SETBP1* (start BP within gene)	Generalized epilepsy of unknown origin, DR	ID	Negative	De novo
BE_LEU_116	Duplication	19p13.3	538568	2268870	1.7	Unknown	*HCN2*	Epilepsy NOS and FS	Learning disabilities, ADHD, facial dysmorphism	Negative	Confirmed in proband, parents NA
US_124	Deletion	20p12.3	8314301	8688028	0.4	Inherited (M+P)	*PLCB1* (start BP within gene)	Epileptic encephalopathy NOS	Profound ID, microcephaly, hypertonia, hyperreflexia more prominent on the left side, squint in left eye	Atrophy on CT brain	NA
PO_W_030	Duplication	20q13.33	61925286	62724437	0.8	Inherited (M)	*KCNQ2, CHRNA4, EEF1A2*	Focal epilepsy of unknown origin	ID, facial dysmorphism	NA	Confirmed in proband, parents NA

ADHD = attention‐deficit/hyperactivity disorder; BP = breakpoint; CC = corpus callosum; CNV = copy number variation; CT = computed tomography; DR = drug resistant; FS = febrile seizures; ID = intellectual disability; M = maternal; MAQ = multiplex amplicon quantification; NA = not available; NOS = not otherwise specified; P = paternal.

Column with candidate genes also includes CNVs including known epilepsy genes that have not been considered pathogenic for various reasons; for example, the direction of the change or the phenotype did not fit to what is reported in the literature, or the CNV was inherited from a parent with unknown affectedness status.

Reported in Ottaviani et al.^43^

In the second category, we identified several interesting candidate genes including a de novo deletion including *STAG1* and a de novo intragenic duplication in *FGF12*. In both genes, only recently have pathogenic SNVs been reported in patients with neurodevelopmental disorders including epilepsy.^24^, ^25^ We further identified a deletion including *SETBP1* associated with Shinzel‐Gieidon syndrome and ID (OMIM 611060) and a duplication including *HCN2,* a gene in which SNVs exerting a gain‐of‐function effect have recently been suggested as a risk factor for genetic generalized epilepsies.^26^ *HCN2* has also previously been associated with febrile epilepsy syndromes; interestingly, the patient carrying this CNV also had a history of febrile seizures.^27^ Other interesting candidate genes located in identified de novo deletions or possibly disrupted by intragenic breakpoints of identified duplications included *FMN2* (also associated with AR mental retardation MIM616193), *CHRM3*,* CSNK1G3*, and *NMT1*, all of which are brain‐expressed and predicted to be intolerant to loss of function (probability of loss‐of‐function intolerance ≥ 0.99) according to the latest gnomAD (http://gnomad.broadinstitute.org/about) constraint metrics (https://www.nature.com/articles/nature19057).

### 4.2 Enrichment analysis

**Figure 2:**
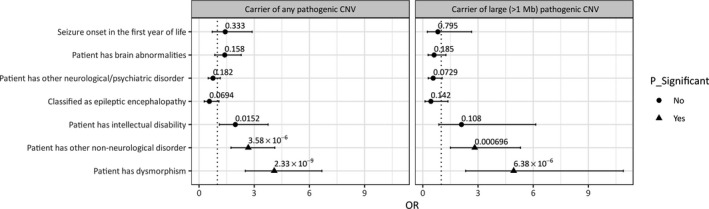
Enrichment analysis. Left panel: Across all patients analyzed in this study, those affected with a pathogenic copy number variation (CNV) were significantly enriched for being comorbid with a nonneurological disorder or dysmorphism. Right panel: Restriction of the analysis to patients carrying large pathogenic CNVs (>1 Mb). These CNV carriers are particularly enriched for nonneurological disorders and dysmorphism. Odds ratios (ORs) significant beyond correction for multiple testing are denoted by triangles

We collected phenotypic information on seven characteristics wherever this specific information was available: neurological or psychiatric disorder (528/956 patients, 55.2%), ID (727/944 patients, 77%), comorbidity with a nonneurological disorder (242/882 patients, 27.4%), facial dysmorphism (209/769 patients, 27.2%), brain abnormalities (288/613 patients, 47%), epilepsy onset before 1 year of age (175/340 patients, 51.5%), and diagnosis of known EE syndrome (238/487 patients, 49%). We compared carriers of pathogenic autosomal CNVs with those who did not carry a pathogenic autosomal CNV. Patients with pathogenic CNVs were significantly enriched for nonneurological disorders (2.68‐fold) and for dysmorphism (4.09‐fold; Figure 2). Beyond the overall pathogenic CNV enrichment, testing for large pathogenic CNVs (>1 Mb) separately showed a more profound and significant fold enrichment of 2.82 and 4.94 for comorbidity with nonneurological disorder and dysmorphism, respectively (Figure 2).

### 4.3 Meta‐analysis

The search identified 4806 citations, of which 59 papers met the inclusion criteria and were included in the systematic review. Overall meta‐analysis showed that in patients with ID without epilepsy, the yield of pathogenic CNVs was 15% (95% confidence interval [CI] = 14‐17), and in patients with psychiatric/neurological disorders without epilepsy, the yield was 8% (95% CI = 5‐12; Figure S3, Table 4). These data were compared with the two subgroups from our cohort: (1) patients with epilepsy and intellectual disabilities, including autistic features, with a yield of 13.5% (95% CI = 9.2‐18.9); and (2) patients with epilepsy and psychiatric/neurological comorbidities, with a yield of 10% (95% CI = 7.9‐11.7). We did not find statistically significant differences for any of these comparisons (*P* values from heterogeneity test were >0.05).

**Table 4:** Comparison of the yield of pathogenic copy number variations in three subgroups of patients from this study with respect to three groups of patients from the literature, analyzed through meta‐analysis

Yield from this study			Yield from meta‐analysis		*P* from test of heterogeneity
	Phenotype (patients, n)	Yield, % (95% CI)	Phenotype	Yield, % (95% CI)	
a	ID + epilepsy (207)	28/207 = 13.5% (9.2‐18.9)	ID	15% (14‐17)	0.4491
b	Psychiatric/neurological comorbidities + epilepsy (528)	53/528 = 10.0% (7.9‐11.7)	Psychiatric/neurological disorders	8% (5‐12)	0.3962
c	Epilepsy‐EE (238)	17/238 = 7.1% (4.2‐11.2)	Epilepsy‐notEE	11% (8‐14)	0.1251

CI, confidence interval; EE, epileptic encephalopathy; ID, intellectual disability.

The yield of pathogenic CNVs in patients with epilepsy‐EE from our cohort (7.1%, 95% CI = 4.2‐11.2) was lower, but not significantly different, than in patients with epilepsy‐notEE from the meta‐analysis (11%, 95% CI = 8‐14; Table 4).

## 5 DISCUSSION

Most epilepsies, especially when beginning in infancy and childhood, have a prominent genetic contribution. Numerous next generation sequencing, whole exome sequencing, and whole genome sequencing studies have been published in recent years uncovering single gene mutations in many epilepsies and epilepsy syndromes. Yet, the contribution of CNVs to the epilepsies, especially those complicated by comorbidities, has been less explored. Most published reports are single‐center studies. The largest sample size was 2454 patients including a large cohort of 1366 patients with genetic generalized epilepsy in addition to 281 patients with rolandic epilepsy and 807 patients with adult focal epilepsy^28^, ^29^; the biggest cohort specifically addressing the epilepsy plus phenotype studied 222 individuals.^4^ The maximum frequency of pathogenic CNVs reported in any of these series was 12%, with a range of 5%‐12%. These studies tended to focus on individuals who were children at the time of testing.^2^, ^5^, ^9^ The importance of rare CNVs has been well recognized in patients with neuropsychiatric disorders including unexplained ID, congenital anomalies, and seizures. Thus, clinical geneticists, pediatric neurologists, and epileptologists commonly request chromosomal array CGH to obtain a genetic diagnosis for patients with such clinical features.

However, CNVs may be seen in healthy control individuals, and determination of the pathogenicity of newly identified CNVs can be challenging. To evaluate the role of pathogenic CNVs and identify possible candidate genes, we investigated the occurrence of CNVs in epilepsy plus, in a cohort among the largest reported to date.^2^, ^3^, ^4^, ^5^, ^9^ Data were collected from eight centers and included both adults and children. Autosomal CNV classification was conducted using a systematic filtering procedure specifically adapted to epilepsy. The workflow was an essential tool to identify, reanalyze, and reinterpret CNVs in this retrospectively collected cohort, in which CNV testing had been performed using different platforms in different laboratories. About 11% of patients with epilepsy plus harbored a pathogenic autosomal CNV. This number reaches 12.7% when we also consider the possibly pathogenic CNVs. Previous similar studies report a diagnostic yield ranging from ~5% to 12%.^2^, ^3^, ^4^, ^5^, ^8^, ^9^ Thus, our result fits at the upper limit of this range, probably mainly due to the “epilepsy plus” phenotype of our cohort and to the application of a standardized workflow. Previously published studies^3^, ^4^, ^8^ that reported similar yields of pathogenic CNVs (9.3%, 8.1%, and 12%, respectively) also examined patients with complex epilepsy including ID. Overall, results from both our and similar previous studies indicate that within the complex phenotype of neurodevelopmental disorders, when seizures are associated with ID or with other neurological and nonneurological comorbidities, there is a higher probability of identifying a pathogenic CNV than in epilepsy alone.

We checked the original classification, where available (138/142), of pathogenic and possibly pathogenic CNVs before and after applying the workflow method we propose here. We found that 7.2% (10/138) of cases were discrepant. The main direction of change was from CNVs (8/10 CNVs) originally classified as of “unknown significance” to “pathogenic” and “possibly pathogenic” (Table S7). This is expected as information about brain‐expressed genes or gene regions associated with epilepsy increases. We have confirmed that reanalysis of existing data over time is essential.

Our study confirms the importance of specific CNVs in epilepsy and broadens some of the associated phenotypic spectra.

Recurrent microdeletions at 1q21.1, 15q11.2, 15q13.3, 16p13.11, and 22q11.21 have been reported as risk factors for GGEs and focal epilepsies.^28^, ^29^ The most frequent CNV identified in our cohort was the 16p13.11 deletion, which accounts for 8.3% of the pathogenic CNVs, supporting a marked relevance in the clinical setting.

We also found several CNVs that included the genes *HNRNPU* (1q44) and *RORB* (9p21.13), both recently associated with epilepsy.^28^, ^30^, ^31^, ^32^ Microdeletions of the 1q43q44 critical region have been associated with ID, dysmorphism, abnormalities of the corpus callosum, and seizures.^30^ This critical region includes *HNRNPU* as the most relevant candidate epilepsy gene. Around 30 point mutations, mainly including truncating, splice‐site, and a few missense variants, in *HNRNPU* have recently been identified in individuals with ID and seizures (Table S6a). In our cohort, five patients carried CNVs mapping to the 1q43q44 critical region, and in addition to the *HNRNPU* gene, in two duplications and one deletion, the chromosomal rearrangement included also the *AKT3* gene, which might contribute to brain abnormalities observed in these patients. Patients with deletions showed dysmorphic features, early onset psychomotor delay, and early onset epilepsy. These data confirm the role of *HNRNPU* in neurodevelopment and epileptogenesis.

Mutations in *RORB* were first reported in a patient with mild ID and partial epilepsy.^31^ More recently, other mutations were identified in patients with neurodevelopmental disorders and mostly GGE, including absence seizures (Table S6b). In our cohort, three patients carried deletions including *RORB* and exhibited ID and generalized epilepsy, including absence seizures with eyelid myoclonia, and autistic features in one patient, supporting a role for *RORB* in GGE and, more broadly, in several neurodevelopmental disorders.

Among the syndromic pathogenic autosomal CNVs, we identified three patients with duplications mapping to the 17p11.2 Potocki‐Lupski syndromic region, which is reciprocal to the Smith‐Magenis deletion syndrome in which epilepsy is often seen.^33^ These three patients had a phenotype consistent with Potocki‐Lupski syndrome; the occurrence of epilepsy supports previous evidence of its presence as a rare feature of 17p11.2 duplications.^5^ Interestingly, we identified five patients with a 7q11.23 deletion containing the Williams‐Beuren region; four of these individuals had Lennox‐Gastaut syndrome, and the fifth (previously reported by Ramocki et al^34^) had a generalized drug‐resistant epilepsy.

CNVs classified as pathogenic only because of large size (Table S4b) represented 27% (33/122) of all the pathogenic CNVs. These CNVs included a large number of genes, but the phenotype of affected individuals was complex and we were unable to identify an association with known genetic syndromes or with candidate epilepsy genes. However, for one individual with EE and a large de novo 13q13.1‐q13.3 deletion, we can suggest that a key gene is *NBEA*, which was reported as a possible EE gene through an in silico prioritization approach^35^ and was recently associated with neurodevelopmental disease with epilepsy.^36^

Four of the CNVs we classified as large and pathogenic were inherited. Interestingly, a duplication on 12q21.31 was inherited from a mother with a family history of autism. Autism has been reported in Decipher in a patient carrying an overlapping duplication (Table S4b). Following our algorithm, we consider these CNVs pathogenic, noting the incomplete penetrance often characterizing neurological and epileptic disorders and because we could not exclude related neurological traits in the transmitting parent.

A possibly pathogenic autosomal CNV was identified in 1.7% of the patients. As well as some known epilepsy genes, discussed in the results section, we propose other genes in these regions that can be considered potential candidates for causing epilepsy, but need further validation. We found a de novo 18q12.3 deletion, which only encompassed the gene *SETBP1*. Heterozygous missense mutations in *SETBP1* cause Schinzel‐Giedion syndrome (OMIM #269150), characterized by severe ID and specific craniofacial features,^37^ wherein seizures also occur.^38^, ^39^ Mutations leading to haploinsufficiency, such as the deletion in our patient, have been reported in association with a distinct neurological syndrome, which includes mild to moderate ID without the typical syndromic craniofacial features.^17^, ^40^, ^41^ The patient in this study only showed severe epilepsy and ID, suggesting that the *SETBP1*‐mutation phenotype may be broader than previously described. One patient had a microdeletion, classified here as possibly pathogenic, which includes *STAG1*, now linked with epilepsy as a cohesinopathy,^24^ and one patient carried a de novo intragenic duplication in *FGF12* in which SNVs have recently been reported in patients with epileptic encephalopathies.^25^

Other interesting candidate genes are highlighted in Table 3 and include *HCN2*,* FMN2*,* CHRM3*,* CSNK1G3*, and *NMT1*.

Eleven patients (1%) in our study cohort had a double hit (including pathogenic and possibly pathogenic CNVs). Here, the CNV burden alone could contribute to the neurodevelopmental phenotype; as shown by Girirajan and colleagues,^42^ children with two or more rare and large CNVs of unknown significance were eight times more likely to have developmental delay compared to controls, possibly by disruption of dosage‐sensitive genes.^42^ We note, however, that our analysis focused only on CNVs with a certain pathogenic meaning and as such gives no insight into the general burden of CNVs per patient.

The enrichment analysis showed a significant association of pathogenic autosomal CNVs with nonneurological disorders and dysmorphism (for both large pathogenic and any pathogenic CNV); large pathogenic CNVs showed a more profound and significant association with dysmorphism and non‐neurological disorders only. An enrichment of CNVs in patients with dysmorphism has been observed in previous studies,^4^ underscoring the importance of testing for CNVs in patients with epilepsy and associated comorbidities. Likewise, results from our data compared to historical controls, from a systematic literature review and meta‐analysis, confirm that the percentages of pathogenic CNVs, when the phenotype includes or excludes epilepsy, do not vary significantly. Thus, although a search for CNV is undoubtedly worthwhile in people with epilepsy plus, it may not be that such CNVs drive only epilepsy, but for patients ascertained through their epilepsy, the presence of additional features points to an elevated likelihood of finding an underlying pathogenic CNV. We hypothesize that although epilepsy as a phenotype does not add a quantitative contribution to the diagnostic yield, its presence could be related to the type, location, and gene content of an underlying pathogenic CNV. Results from our data, comparing patients with epilepsy‐EE versus historical controls with epilepsy‐notEE, showed a nonsignificantly lower yield of pathogenic CNVs in patients with EE, raising a possible hypothesis that when epilepsy manifests as EE, the likelihood of finding a pathogenic CNV decreases and that EE is more often the consequence of single gene mutations.

Our study has limitations beyond its retrospective structure. The filtering workflow used allowed us to obtain a systematic classification of the large number of CNVs examined, but we recognize it is not perfect and might not accurately classify CNV mapping to hypervariable chromosomal regions. Pathogenic CNVs could be missed due to filtering out of small CNVs, misclassification of abnormalities, or an incomplete list of genes associated with epilepsy (new epilepsy‐related genes continue to be reported). We excluded the sex chromosomes from the CNV calling and subsequent analysis, because copy number calling from these chromosomes is prone to false‐positive calls and might inflate the reported frequencies of diagnostically relevant CNVs as the X chromosome in particular has been associated with neurodevelopmental disorders. Also, recessive disease cannot be ruled out with this type of analysis unless the second allele is studied with another approach.

In conclusion, we highlight the pathogenic causative role of autosomal CNVs in almost 11% of patients (and up to 12.7% when also considering the possibly pathogenic) with unexplained epilepsy with comorbidities and reiterate the concept that CNVs should be sought in patients with seizures especially when associated with other neurological and nonneurological conditions.

This study opens new perspectives for a better understanding and evaluation of CNVs identified in patients with epilepsy plus. We show that the reinterpretation of preexisting data using an adapted workflow can highlight new findings, and we recommend periodic systematic review of preacquired genetic data, as new methods and data become available. The workflow used here, specifically designed for epilepsy, can be used to homogenize data from different cohorts often collected at different times. Establishing the causative role of some CNVs can be challenging, especially when the CNV is not associated with a known syndrome, or similar CNVs may not be of the same size, might include different genes, and not have familial segregation data available to help interpretation. Bespoke, disease‐specific algorithms may assist in assignment of CNVs to diagnostic categories that are more definitive than either “possibly pathogenic” or “of unknown significance.” There remain CNVs whose role will only be clarified by increasing the number of cases studied, functional studies, and continued exchange between clinicians and laboratory scientists. This study represents the first project of a newly formed and growing international consortium for CNVs in epilepsy (EpiCNV), in which large‐scale data aggregation and sharing will be utilized as a new tool for CNV and gene identification in the epilepsies.

## DISCLOSURE OF CONFLICT OF INTEREST

A.C. has received honoraria from Eisai for participating in on an advisory board and as a speaker. B.C. has received honoraria for consulting and advisory board services from Brabant Pharma, Zogenix, and Novartis. J.H.C. is involved in clinical trials for GW Pharma and Zogenix; has received research grants from Vitaflo, NIHR, Action Medical Research, SPARKS, and the European Union; has been part of advisory boards for Takeda, Shire, UCB, and Eisai; and has given lectures for Shire and Zogenix; all honoraria and funds were given to the department. L.L. has received honoraria from Zogenix, Livanova, Shire, Takeda, UCB, and Novartis. P.S. has received speaker honoraria and travel grants from Eisai, Kolfarma, FB Health, and Zogenix. C.M. is an associate editor of *Epileptic Disorders* and has received speaker honoraria from SOBI (Swedish, Orphan Biovitrum). None of the other authors has any conflict of interest to disclose. We confirm that we have read the Journal's position on issues involved in ethical publication and affirm that this report is consistent with those guidelines.

## AUTHOR CONTRIBUTIONS

All authors made substantial contributions to the conception and design of the study, acquisition of data, or analysis and interpretation of data; drafting of the article or revising it critically for important intellectual content; or appropriate investigation of accuracy and integrity of some part of the manuscript, in addition to giving final approval of the submitted version. The individual contribution for the manuscript of each author is as follows. A.C.: analysis and interpretation of data, manuscript preparation, revising manuscript critically for important intellectual content; E.C.: analysis and interpretation of data, manuscript preparation, revising manuscript critically for important intellectual content; H.S.: analysis and interpretation of data, manuscript preparation, revising manuscript critically for important intellectual content; E.Sa.: analysis and interpretation of data, manuscript preparation, revising manuscript critically for important intellectual content; V.C.: analysis and interpretation of data, manuscript preparation, revising the manuscript; D.L.: analysis and interpretation of data, revising the manuscript; T.Dj.: acquisition and analysis of data, revising the manuscript; M.B.‐G.: acquisition of data, revising the manuscript; B.C.: acquisition of data, revising the manuscript; J.H.C.: acquisition of data, revising the manuscript; T.De.: analysis of data, revising the manuscript; S.D.M.: analysis and interpretation of data; T.Do.: acquisition of data, revising the manuscript; R.G.: study concept, revising manuscript critically for important intellectual content; D.H.‐Z.: acquisition of data, revising the manuscript; F.K.; acquisition of data, revising the manuscript; L.L.: acquisition of data, revising the manuscript; N.L.: acquisition of data, revising the manuscript; J.R.L.: acquisition of data, revising the manuscript; E.L.: analysis and interpretation of data; F.M.: acquisition of data, revising the manuscript; H.C.M.: acquisition and analysis of data, revising the manuscript; D.M.: analysis and interpretation of data, revising the manuscript; P.N.: acquisition of data, revising the manuscript; A.P.: analysis and interpretation of data, revising the manuscript; A.S.S.: acquisition of data, revising the manuscript; P.S.: acquisition of data, revising the manuscript; E.Sz.: acquisition of data, revising the manuscript; A.T.: acquisition of data, revising the manuscript; J.R.V.: acquisition of data, revising the manuscript; H.V.E.: acquisition of data, revising the manuscript; W.V.P.: acquisition of data, revising the manuscript; J.J.W.: acquisition of data, revising the manuscript; S.W.: acquisition and analysis of data, revising the manuscript; F.Z.: acquisition of data, revising the manuscript; P.D.J.: study concept and supervision, manuscript preparation, revising manuscript critically for important intellectual content; S.M.S.: study concept and supervision, interpretation of data, manuscript preparation, revising manuscript critically for important intellectual content; C.M.: study concept and supervision, interpretation of data, manuscript preparation, revising manuscript critically for important intellectual content.

## ETHICS

This study was approved by the ethics committees of the participating centers. Written informed consent was provided by each patient, or the parent or guardian of each patient, as appropriate.

## APPENDIX 1 COLLABORATORS

### EuroEPINOMICS‐RES Consortium

Anna‐Elina Lehesjioki (Folkhälsan Institute of Genetics Neuroscience Center and Research Programs Unit Molecular Neurology, University of Helsinki, Helsinki, Finland), Dana Craiu (Carol Davila University of Medicine Bucharest, Department of Clinical Neurosciences [No. 6], Pediatric Neurology Clinic, Alexandru Obregia Hospital, Bucharest, Romania), Tiina Talvik (Tartu University Hospital, Children's Clinic, Tartu, Estonia; Department of Pediatrics, University of Tartu, Tartu, Estonia), Hande Caglayan (Department of Molecular Biology and Genetics, Bogaziçi University, Istanbul, Turkey), Jose Serratosa (Neurology Laboratory and Epilepsy Unit, Department of Neurology, Instituto de Investigatión Sanitaria‐Fundación Jiménez Díaz, Autonomous University of Madrid, Madrid, Spain; IIS–Jiménez Díaz Foundation and Center for Biomedical Research in the Network of Rare Diseases, Madrid, Spain), Katalin Sterbova (Department of Child Neurology, 2nd Faculty of Medicine, Charles University, Motol Hospital, Prague, Czech Republic), Rikke S. Møller (Danish Epilepsy Center, Dianalund, Denmark and Institute for Regional Health Services, University of Southern Denmark, Odense, Denmark), Helle Hjalgrim (Danish Epilepsy Center, Dianalund, Denmark and Institute for Regional Health Services, University of Southern Denmark, Odense, Denmark), Holger Lerche (Department of Neurology and Epileptology, Hertie Institute for Clinical Brain Research, University of Tübingen, Tübingen, Germany), Yvonne Weber (Department of Neurology and Epileptology, Hertie Institute for Clinical Brain Research, University of Tübingen, Tübingen, Germany), Ingo Helbig (Division of Neurology, Children's Hospital of Philadelphia, Philadelphia, Pennsylvania and Department of Neuropediatrics, University Medical Center Schleswig‐Holstein, Kiel, Germany), Sarah von Spiczak (Department of Neuropediatrics, University Medical Center Schleswig‐Holstein, Kiel, Germany).

### EpiCNV Consortium (info@epicnvsconsortium.net)

Carmen Barba (Meyer Children's Hospital, Florence, Italy), Anneleen Bogaerts (Center for Human Genetics, University Hospitals Leuven, Leuven, Belgium), Antonella Boni (IRCCS Bellaria Hospital, Bologna, Italy), Elisabeth Caruana Galizia (Department of Clinical and Experimental Epilepsy, University College London, London, UK), Sara Chiari (Meyer Children's Hospital, Florence, Italy), Claudia Clementella (Meyer Children's Hospital, Florence, Italy), Gianpiero Di Gacomo (Meyer Children's Hospital, Florence, Italy), Annarita Ferrari (IRCCS Stella Maris, Calambrone, Pisa, Italy), Silvia Guarducci (Meyer Children's Hospital, Florence, Italy), Sabrina Giglio (Meyer Children's Hospital, Florence, Italy), Philip Holmgren (Department of Medical Genetics, University of Antwerp and Antwerp University Hospital, Antwerp, Belgium), Costin Leu (University College London, London, UK), Francesco Mari (Meyer Children's Hospital, Florence, Italy), Federico Melani (Meyer Children's Hospital, Florence, Italy), Francesca Novara (Department of Molecular Medicine, University of Pavia, Pavia, Italy), Marilena Pantaleo (Meyer Children's Hospital, Florence, Italy), Elke Peeters (Department of Neurology, University Hospitals Leuven, Leuven, Belgium), Tiziana Pisano (Meyer Children's Hospital, Florence, Italy), Anna Rosati (Meyer Children's Hospital, Florence, Italy), Josemir Sander (Department of Clinical and Experimental Epilepsy, University College London, London, UK), Natasha Schoeler (Department of Clinical and Experimental Epilepsy, University College London, London, UK), Pawel Stankiewicz (Department of Medical Genetics, Institute of Mother and Child, Warsaw, Poland), Salvatore Striano (Federico II University, Naples, Italy), Arvid Suls (Center for Medical Genetics, University of Antwerp, Antwerp, Belgium), Monica Traverso (Department of Neurosciences, Rehabilitation, Ophthalmology, Genetics, and Maternal and Child Health, University of Genoa, G. Gaslini Institute, Genoa, Italy), Geert Vandeweyer (Department of Medical Genetics, University of Antwerp and Antwerp University Hospital, Antwerp, Belgium), Anke Van Dijck (Department of Medical Genetics, University of Antwerp and Antwerp University Hospital, Antwerp, Belgium), Orsetta Zuffardi (Department of Molecular Medicine, University of Pavia, Pavia, Italy).
