# Supplementary material for: Diagnostic implications of genetic copy number variation in epilepsy plus
Source: Epilepsia. 2019 Mar 13;60(4):689–706. doi: 10.1111/epi.14683 (PMC6488157; doi:10.1111/epi.14683)
Supplement: Supplementary file 1 [file EPI-60-689-s001.docx]

**SUPPLEMENTARY MATERIALS**

1. Supplementary Methods
   1. Array platforms used
   2. CNV annotation
   3. Detailed description of the stepwise procedures for CNV classification using the workflow
   4. Confirmation of possibly pathogenic autosomal CNVs using Multiplex Amplicon Quantification (MAQ)
   5. Phenotype enrichment analysis
   6. Systematic Review and Meta-analysis
   7. Selection of epilepsy candidate genes from Possibly Pathogenic CNVs
2. Supplementary tables and figures
   1. Table S.1: List of the different array platforms used
   2. Table S.2: List of the genes associated with epilepsy used for annotation
   3. Table S.3: General results from pathogenic and possibly CNVs
   4. Table S.4a: Non-recurrent pathogenic CNVs associated with known genetic syndromes
   5. Table S.4b: CNVs classified as pathogenic because of size (> 3 Mb or <3 and > 1 Mb and de novo)
   6. Table S.5: patients with CNVs including a) *HNRNPU* and b) *RORB*
   7. Table S.6: literature data on a) *HNRNPU* and b) *RORB*
   8. *Table S7*: CNVs having a different classification before/after application of the workflow
   9. Figure S.1: Plot of all CNVs per sample across all samples included in the study (N=1097)
   10. Figure S.2: MAQ electropherogram
3. References

**1. Supplementary Methods**

*a. CNV platforms used.*

Array-CGH platforms used to genotype the cohort were: Agilent105K, Agilent180K, Agilent244K, Agilent44B, Agilent44K, Agilent60K, Customized Agilent 180K with additional gene coverage, Customized Agilent AMADID060585, Illumina HumanCNV370duo, Illumina HumanCNV370quad, Illumina HumanCyto12v1.0, Illumina HumanCyto12v2.0, Illumina HumanCyto12v2.1, Illumina HumanCyto12v2.1_L and NimbleGen135K.
See Supplementary Table S.2 for more details

*b. CNV gene annotation*

We annotated the following sets of genes and loci: all protein-coding RefSeq hg19 genes defined by the transcript (accessed December 2015), known epilepsy-associated hot spot loci^1^, genes covered by a presumed benign CNV in 2,647 healthy subjects from diverse ethnicities ^2^ , ASD-related genes ^3^ , brain-expressed genes ^4^ and an in-house list of epilepsy candidate genes from the literature (Supplementary Table S.2). Genes with ambiguous positions (haplotype-dependent) in the RefSeq hg19 gene list were excluded from the analysis. All annotations were conducted using customized Perl and R- scripts, available upon request.

*c. Detailed description of the stepwise procedures for CNV classification using the workflow*

After quality control the remaining CNVs were classified manually using a flowchart. This workflow considered three different steps:

1) CNVs already reported in the paper from Zarrei et al.^2^ reporting on CNVs in control populations were considered as common and classified as ‘benign’ CNVs, and thus filtered out^2^. All the remaining CNVs were considered “rare” and entered the second step.

2) CNVs fitting into one of the following categories were classified as ‘pathogenic’: (a) CNVs with ≥80% overlap with genomic regions where the association with epilepsy has already been established^5-9^ (b) CNV with a size ≥3 Mb and a least 1 brain expressed gene or (c) CNV <3 Mb but >1 Mb and with *de novo* occurrence (following the American College of Medical Genetics guidelines^10^). We compared the phenotype of our patients with the reported phenotype for a given CNV using OMIM and available literature. CNVs that overlapped those reported in patients with similar phenotypes were deemed ‘pathogenic CNVs’; the remaining CNVs were considered CNVs of ‘uncertain significance’ and entered the third step.

3) The uncertain CNVs were analysed by gene content. We searched for the presence of genes currently known to be associated with an epilepsy phenotype using an annotated gene list curated by ourselves (Supplementary Table S.2) and by manually going though databases such as OMIM, Genecards and PubMed. By comparing the variant described in the literature with those observed in our cases, we classified a CNV as pathogenic if the CNV we observed (deletion or duplication) corresponded to what has been observed in the literature (gain or loss of function or both). If the CNV included a gene associated with epilepsy by our classification, but the CNV type and/or the phenotype was not the same as observed in association with the phenotype reported in the literature, we considered the inheritance. If the CNV was proven to occur *de novo*, we also classified the CNV as “pathogenic”. If the CNV was inherited and we had no phenotypic information on the parents, or if the segregation was unknown, we conservatively classified the CNV as possibly pathogenic.

When a CNV did not encompass a gene associated with epilepsy, we considered the function and brain expression of all the genes included studied using the GeneCards database (<http://www.genecards.org/>), GTEX portal (<http://www.gtexportal.org/home/>) and Pinto et al^4^, and again considered inheritance. If at least one of the included genes was highly expressed in the brain and/or a function within the central nervous system was reported, and the CNV was not inherited, the CNV was classified as possibly pathogenic. Otherwise it was considered as a CNV of unknown significance.

By using this workflow, all CNVs were classified into four groups: benign, pathogenic, possibly pathogenic and of unknown significance.

*d. Confirmation of possibly pathogenic autosomal CNVs with Multiplex Amplicon Quantification (MAQ)*

To validate the possibly pathogenic CNVs identified using array-CGH or SNP-array, we used a locally-developed technique for multiplex amplicon quantification (MAQ, [https://www.agilent.com/en/products/next-generation-sequencing/amplicon-target-amplification-(multiplicom)/maq-overview](https://www.agilent.com/en/products/next-generation-sequencing/amplicon-target-amplification-(multiplicom)/maq-overview) ). This assay comprises multiplex PCR amplification of fluorescently-labelled target and reference amplicons followed by fragment analysis on the ABI 3730 DNA Analyzer. The target amplicons are located in the CNV region whereas the reference amplicons are randomly located on different chromosomes in region with the normal copy number (two). The comparison of normalized peak areas for the test individual and the average of control individuals yields target amplicon doses indicating the copy number of the target amplicon (using the MAQ-s software, Agilent, [https://www.agilent.com/en/products/next-generation-sequencing/amplicon-target-amplification-(multiplicom)/maq-s](https://www.agilent.com/en/products/next-generation-sequencing/amplicon-target-amplification-(multiplicom)/maq-s)), (see Supplementary Figure S1 and S2).

*e. Phenotype enrichment analysis*

Beyond the individual identification and classification of CNVs, we investigated whether patients affected by a pathogenic autosomal CNV where enriched for specific features. We collected phenotype information on six features for sub-fractions of the cohort for whom this information was available and used Fisher’s exact test to identify specific enrichment among patients carrying a pathogenic CNV compared to the non-carriers in the cohort. We were able to collect the information for whether I) a patient is or is not comorbid for a non-neurological disorder for 882 patients; II) a patient is or is not comorbid for a neurological or psychiatric disorder for 956 patients; III) a patient is or is not comorbid for ID for 944 patients; IV) a patient is or is not comorbid for facial dysmorphisms for 769 patients; V) a patient is or is not comorbid for structural brain abnormalities for 613 patients; VI) a patient’s seizures have or have not an onset before one year of age for 340 patients; and VII) a patient’s epilepsy diagnosis is or is not classified as epileptic encephalopathy for 487 patients.

*f. Systematic Review and Meta-analysis*

To obtain control groups, we used published data. We conducted a systematic review of the literature for papers reporting the yield of pathogenic CNVs in patients with intellectual disability (without epilepsy), psychiatric/neurological comorbidities (without epilepsy) and epilepsy (without epileptic encephalopathy).

We performed a MEDLINE (via PubMed) search from 1st January 2000 to 6 th September 2018, using the following search strategy: copy number variation"[Title/Abstract] OR "copy number variations"[Title/Abstract] OR "DNA copy number variations"[MeSH Terms] OR "microduplications"[Title/Abstract] OR "microdeletions"[Title/Abstract] OR "array comparative genomic hybridization"[Title/Abstract] OR "chromosomal microarray"[Title/Abstract]). We also used these filters: English language, human studies and age >5 years, to exclude patients in which epilepsy had not yet occurred but might manifest later.

We reviewed titles and abstracts of citations identified through literature search, and selected those including patients with the above mentioned phenotypes.

We then retrieved the full text of all these selected studies and included papers meeting the following criteria:

1. CNV identified with Array-CGH or SNP-Array;
2. Sample size more than 50 patients;
3. Patient phenotypes including: a)intellectual disability, b) psychiatric/neurological disorders, c) epilepsy without epileptic encephalopathy (EE).

We excluded papers examining genetically isolated populations. We performed a meta-analysis of proportions by grouping papers according to patients’ phenotypes, i.e. intellectual disability psychiatric/neurological disorders and epilepsy. First, to establish the variance of raw proportions we applied a Freeman-Tukey transformation. Second, to incorporate heterogeneity (which was anticipated among the included studies), transformed proportions were combined using random effects models. Finally, the pooled estimates were back-transformed. Heterogeneity across studies was evaluated using the Cochran Q test. Thus, the results were presented as pooled proportions (%) with 95% confidence intervals (Figure S.3). The search identified 4806 citations from PubMed, of which 59 papers met the inclusion criteria and were included in the systematic review^5-9,11-63^.

g. *Selection of epilepsy candidate genes from possibly pathogenic CNVs*

All genes included in the possibly pathogenic autosomal CNV were annotated and prioritized according to following criteria: (1) absence in the healthy population^2^, (2) expression levels in the central nervous system (Pinto et al., 2014^4^; GTEX database), (3) possible known association with a neurodevelopmental phenotype (e.g. seizures, autism or intellectual disability) and (4) tolerance/intolerance to loss-of-function with respect to the deletions (probability of loss-of-function intolerance (pLI) score from Exome Aggregation Consortium-ExAC-database). With this approach we selected for each *de novo* CNV one or two of the included, brain-expressed, genes that have already been associated with neurodevelopment and best fit the criteria for epilepsy candidate gene. For inherited CNVs, or for CNVs with an unknown inheritance, we selected known epilepsy related genes (table 3).

| **City** | **Institution** | **# of patients** | **Platform** |
| --- | --- | --- | --- |
| London | North East Thames Regional Genetics Laboratory Services, Great Ormond Street Hospital for Children NHS Foundation Trust, London, UK | 299 | NimbleGen 135K whole genome v3.0 array chip |
| Antwerp | Department of Medical Genetics, University and University Hospital Antwerp, Antwerp, Belgium | 165 | HumanCyto12v1.0; HumanCyto12v2.0; HumanCyto12v2.1; HumanCyto12v2.1_L HumanCNV370quad, HumanCNV370duo |
| Antwerp | Neurogenetics Group, Center for Molecular Neurology, VIB, Antwerp, Belgium | 28 | HumanCNV370quad |
| Leuven | Center for Human Genetics, University Hospitals Leuven, Herestraat 49, 3000 Leuven, Belgium | 261 | Agilent 105K, Agilent 180K |
| Genoa | Neurogenetic Laboratory, DiNOGMI, G Gaslini Institute, Genova, Italy | 27 | Genome CGH Microarray Kit 4 x 44K (Agilent) |
| Seattle | Division of Genetic Medicine, Department of Pediatrics, University of Washington, Seattle, USA | 275 | Agilent 4x180 catalog Array; Custom Agilent 4x180+genes, Custom Agilent AMADID 060585 |
| Florence | Medical Genetics Unit, Meyer Children's University Hospital Florence, Italy. | 157 | Agilent 44B, Agilent 60K, Agilent 105K, Agilent 244K, Agilent 180K |
| Warsaw | Department of Medical Genetics, Instituite of Mother and Child, Warsaw, Poland | 43 | NimbleGen 385K ChrX; NimbleGen 3x720K ChrX Custom; CGH-array: V7.4 (105K), Agilent Technologies; CGH-array: V8.1 (180K), Agilent Technologies |
| **total** |  | **1255** |  |

**2. Supplementary tables and figures:**

*a. Table S.1:* Different array platforms used, institution where the genetic test was performed and
 numbers of individuals per centre.

*b. Table S.2:* List of the genes associated with epilepsy used for annotation

| **Gene** | **Gene/Locus MIM number** | **Phenotype MIM number** | **References** |
| --- | --- | --- | --- |
| ABAT | 137150 | 613163 | Besse et al, 2016 |
| ADGRV1 | 602851 | 604352 | Nakayama et al., 2002 |
| ALDH7A1 | 107323 | 266100 | Mills et al., 2010 |
| ALG13 | 300776 | 300884 | de Ligt et al., 2012 |
| ARFGEF2 | 605371 | 608097 | Sheen et al., 2004 |
| ARHGEF9 | 300429 | 300607 | Shimojima et al., 2011 |
| ARX | 300382 | 308350, 200215, 300419 | Strømme et al., 2002 |
| ATN1 | 607462 | 125370 | Koide et al., 1994 |
| ATP1A2 | 182340 | 104290, 602481 | Vanmolkot et al., 2003 |
| ATP1A3 | 182350 | 614820 | Heinzen et al 2012 |
| ATP6AP2 | 300556 | 300423 | Gupta et al., 2015 |
| CACNA1A | 601011 | 617106, 141500 | Jouvenceau et al., 2001 |
| CACNA1G | 604065 | 616795 | Yalç?n et al., 2012 |
| CACNA1H | 607904 | 611942 | Chen et al., 2003 |
| CASR | 601199 | 612899 | Kapoort et al., 2008 |
| CDKL5 | 300203 | 300672 | Weaving et al., 2004 |
| CHRNA2 | 118502 | 610353 | Aridon et al., 2006 |
| CHRNA4 | 118504 | 600513 | Steinlein et al., 1995 |
| CHRNA7 | 118511 | 118511 | Helbig et al., 2009 |
| CHRNB2 | 118507 | 605375 | Phillips et al., 2001 |
| CLN3 | 600580 | / | Licchetta et al., 2015 |
| CLN5 | 608102 | 256731 | Savukoski et al., 1998 |
| CLN6 | 606725 | 601780 | Arsov et al., 2011 |
| CLN8 | 607837 | 610003 | Striano et al., 2007 |
| CNTN2 | 190197 | 615400 | Stogman et al., 2013 |
| CNTNAP2 | 604569 | 610042 | Penagarikano et al., 2011 |
| COL4A1 | 120130 | 611773, 175780 | Hino-Fukuyo et al., 2016 |
| CPA6 | 609562 | 614417, 614418 | Salzmann et al., 2012 |
| CSTB | 601145 | 254800 | Lehesjoki et al., 1991 |
| CTSD | 116840 | 610127 | Siintola et al., 2006 |
| D2HGDH | 609186 | 600721 | Struys et al., 2005 |
| DCX | 300121 | 300067 | Matsumoto et al., 2001 |
| DNAJC5 | 611203 | 162350 | Noskova et al., 2011 |
| DNM1 | 602377 | 616346 | Appenzeller S, et al., EuroEPINOMICS-RES consortium , 2014 |
| EHMT1 | 607001 | 610253 | Kleefstra T et al., 2009 |
| EMX2 | 600035 | 269160 | Brunelli et al., 1996 |
| EPM2A | 607566 | 254780 | Minassian et al., 1998 |
| FKRP | 606596 | 613153 | Kondo-lida et al., 1999 |
| FKTN | 607440 | 253800 | Kondo-lida et al., 2009 |
| FLNA | 300017 | 300049 | Parrini et al., 2006 |
| FMR1 | 309550 | 300624 | Berry-Kravis et al., 2002 |
| FOLR1 | 136430 | 613068 | Al-Baradie et al., 2014 |
| FOXG1 | 164874 | 613454 | Mencarelli et al., 2010 |
| GABRA1 | 137160 | 615744 | [Maljevic et al., 2006](http://omim.org/geneMap/20/297?start=-3&limit=10&highlight=297) |
| GABRB1 | 137190 | 617153 | Allen at al., Epi4K Consortium and Epilepsy Phenome, 2013 |
| GABRB3 | 137192 | 617113 | Tanaka et al., 2008 |
| GABRG2 | 137164 | 611277 | Audenaert et al., 2006 |
| GNAO1 | 139311 | 615473 | Nakamura et al., 2013 |
| GOSR2 | 604027 | 614018 | Boisse-lomax et al., 2013 |
| GPHN | 603930 | 603930 | Dejanovic, et al., 2013 |
| GPR56 | 604110 | 606854 | Parrini et al., 2009 |
| GRIN1 | 138249 | 614254 | Lemke et al., 2016 |
| GRIN2A | 138253 | 245570 | Endele et al., 2012 |
| GRIN2B | 138252 | 616139, 613970 | Lemke et al., 2014 |
| HCN2 | 602781 | / | Dibbens et al., 2010 |
| HEXA | 606869 | 272800 | Paw et al., 1990 |
| HNRNPU | 602869 | 602869 | Allen at al., Epi4Kconsortium, 2013; de Kovel et al, 2016 |
| IMPA2 | 605922 | / | Nakayama et al., 2004 |
| IQSEC2 | 300522 | 309530 | Zerem et al., 2016 |
| KCNA1 | 176260 | 160120 | Zuberi et al., 1999 |
| KCNA2 | 176262 | 616366 | Pena and Coimbra et al., 2015 |
| KCNB1 | 600397 | 616053 | Torkamani et al., 2014 |
| KCNJ10 | 602208 | 612780 | Lenzen et al., 2005 |
| KCNJ11 | 600937 | 606176 | Battaglia et al., 2012 |
| KCNMA1 | 600150 | 609446 | Du et al., 2005 |
| KCNQ2 | 602235 | 613720, 121200 | Singh et al., 1998 |
| KCNQ3 | 602232 | 121201 | Charlier et al., 1998 |
| KCNT1 | 608167 | 614959, 615005 | Barcia et al., 2012 |
| KCTD7 | 611725 | 611726 | Krabichler et al., 2012 |
| LARGE | 603590 | 608840 | Longman et al., 2003 |
| LGI1 | 604619 | 600512 | Berkovic et al., 2004b |
| LIS1 | 601545 | 607432 | Cardoso et al., 2002 |
| MAGI2 | 606382 | / | Marshall et al 2008 |
| MECP2 | 300005 | 300673, 300055, 312750 | Hampson et a., 2000 |
| MEF2C | 600662 | 613443 | Le Meur et al., 2010 |
| MFSD8 | 611124 | [610951](http://www.omim.org/entry/616638) | Siintola et al., 2007 |
| MTOR | 601231 | 616638 | Smith et al., 2015 |
| NEDD4L | 606384 | 617201 | Dibbens et al., 2007 |
| NF1 | 162200 | 162200 | Carranzana at al., 1993 |
| NF2 | 607379 | 101000 | Ruggieri et al., 2005 |
| NHLRC1 | 608072 | 254780 | Chan et al., 2003 |
| NIPA1 | 608145 | 600363 | Svenstrup et al., 2011 |
| NRXN1 | 600565 | 614325 | Zweier et al., 2009; Harrison et al., 2009 |
| PAFAH1B1 | 601545 | 607432 | Cardoso et al., 2002 |
| PAX6 | 607108 | 106210 | Glaser et al., 1994 |
| PCDH19 | 300460 | 300088 | Dibbens et al., 2008 |
| PDYN | 121240 | 610245 | Stögmann et al., 2002 |
| PHF6 | 300414 | 301900 | Lower et al., 2002 |
| PI12 | 602445 | 604218 | Hagen et al., 2011 |
| PLCB1 | 607120 | 613722 | Kurian et al., 2010 |
| PNKP | 605610 | 613402 | Shen et al., 2010 |
| PNPO | 603287 | 610090 | Pearl et al., 2012 |
| POLG | 174763 | 607459 | Anagnostou et al., 2016 |
| POMGNT1 | 606822 | 253280 | Beltran Valero de Bernabé et al., 2002 |
| POMT1 | 607423 | 236670 | van Reeuwijk et al., 2006 |
| POMT2 | 607439 | 613150 | van Reeuwijk et al., 2005 |
| PPT1 | 600722 | 256730 | Vesa et al., 1995 |
| PRICKLE1 | 608500 | 612437 | Bassuk et a., 2008 |
| PRICKLE2 | 608501 | / | Tao et al., 2011; Sandford et al., 2016; Mahajan and Bassuk et al., 2016 |
| PRNP | 176640 | 137440 | Valadão et al., 2014 |
| PRRT2 | 614386 | 602066, 128200, 605751 | Chen et al., 2012 |
| RELN | 600514 | 257320, 616436 | Zaki et al., 2007 |
| SCARB2 | 602257 | 254900 | Berkovic et al., 2008 |
| SCN1A | 182389 | 607208, 604403, 609634 | Wallace et al., 2003, Escayg et al., 2000 |
| SCN1B | 600235 | 604233 | Wallace et al. 1998 |
| SCN2A | 182390 | 613721, 607745 | Striano et al., 2006 |
| SCN8A | 600702 | 614558, 617080 | Martin et al., 2007 |
| SLC12A5 | 606726 | 616645 | Stodberg et al., 2015 |
| SLC13A5 | 608305 | 615905 | Thevenon et al., 2014 |
| SLC1A2 | 600300 | 617105 | Epi4K Consortium., Am. J. Hum. Genet 2016 |
| SLC25A12 | 603667 | 612949 | Falk et al., 2014 |
| SLC25A22 | 609302 | 609304 | Molinari et al., 2005 |
| SLC2A1 | 138140 | 606777, 612126 | Striano et al., 2012 |
| SLC35A2 | 314375 | 300896 | Kodera et al., 2013 |
| SLC35A3 | 605632 | 615553 | Edvardson et al., 2013 |
| SLC6A8 | 300036 | 300352 | Hahn et al., 2002 |
| SLC9A6 | 300231 | 300243 | Garbern et al., 2010 |
| SPTAN1 | 182810 | 613477 | Tohyama et al., 2008 |
| ST3GAL5 | 604402 | 609056 | Simpson et al., 2004 |
| STRADA | 608626 | 611087 | Bi et al. , 2016 |
| STX1B | 601485 | 616172 | Schubert et al., 2014 |
| STXBP1 | 602926 | 612164 | Deprez et al., 2010 |
| SYN1 | 313440 | 300491 | Garcia et al., 2004 |
| SYNGAP1 | 612621 | 603384 | Mignot C, et al., 2016 |
| SYP | 313475 | 300802 | Tarpey et al., 2009 |
| TBC1D24 | 613577 | 615338, 605021 | Falace et al., 2010 |
| TCF4 | 602272 | 610954 | Amiel et al. , 2007 |
| TK2 | 188250 | 609560 | Galbiati et al., 2006 |
| TPP1 | 607998 | 204500 | Sleat et al., 1997 |
| TSC1 | 605284 | 191100 | Dabora et al., 2001 |
| TSC2 | 191092 | 613254 | Dabora et al., 2001 |
| TUBA1A | 602529 | 611603 | Poirier et al., 2007 |
| TUBB2B | 612850 | 615752 | Jaglin et al.,2009 |
| UBE3A | 601623 | 105830 | Buiting et al., 2016 |
| VLDLR | 192977 | 224050 | Boycott et al., 2005 |
| ZEB2 | 605802 | 235730 | Wakamatsu et al., 2001 |

*c. Table S.3:* General results from pathogenic and possibly pathogenic CNVs

| **CNV** | **Number of CNVs** | **Number of individuals with at least one CNVs** | **% individuals with 1 CNV (total 1097 individuals)** |
| --- | --- | --- | --- |
| Pathogenic duplications | 33 | 32 | 2.92% |
| Possibly pathogenic duplications | 10 | 10 | 0.91% |
| Pathogenic deletions | 89 | 88 | 8.02% |
| Possibly pathogenic deletions | 10 | 9 | 0.82% |
| Pathogenic CNVs (duplication + deletion) | 122 | 120 | **10.94%** |
| Possibly pathogenic CNVs (duplication + deletion) | 20 | 19 | 1.73% |
| Combined | 142 | 139 | 12.67% |
| Double hit (pathogenic and possibly pathogenic) |  | 11 | 1.0% |

*d. Table S.4a:* Autosomal CNVs related to a genetic OMIM syndrome with neurological symptoms in which epilepsy can feature

CNVs mapping in regions involved in well-characterized genetic syndromes frequently associated with epilepsy. The OMIM reference is given.

| **Individual** | **CNVs Type** | **Chr region** | **Start** | **Stop** | **Size (Mb)** | **Inheritance** | **Syndrome** | **OMIM/Reference** |
| --- | --- | --- | --- | --- | --- | --- | --- | --- |
| IT_FLO_014 | Deletion | 2q22-q23 | 141060000 | 151150000 | 10.1 | *De Novo* | Mowat-Wilson syndrome | # 235730 |
| IT_FLO_103 | Duplication | 2q24.3 | 164713623 | 168145606 | 3.4 | *De Novo* | 2q24.3 duplication | [Yoshitomi et al. 2015](http://omim.org/entry/607208) |
| UK_L_162 | Duplication | 3q29 | 195459696 | 197464256 | 2 | Unknown | 3q29 duplication syndrome | #611936 |
| IT_FLO_109 | Deletion | 4p16.3 | 71352 | 1715428 | 1.6 | *De Novo* | Wolf-Hirschhorn deletion syndrome | #194190 |
| IT_FLO_061 | Duplication | 4p16.3-p13 | 72000 | 42129072 | 42 | *De Novo* | 4p16.3 microduplication syndrome | Palumbo et al, 2015 |
| BE_ANT_062 | Deletion | 7q11.23 | 72722981 | 76409795 | 3.7 | *De Novo* | Contains Williams Beuren syndrome (WBS) region. | #194050 |
| PO_W_018 | Deletion | 7q11.23 | 75165479 | 76823728 | 1.7 | Inherited (M) | Contains Williams Beuren syndrome (WBS) distal region. | #194050/ Ramocki et al. 2010 |
| IT_FLO_002 | Deletion | 7q11.23 | 72716349 | 74138349 | 1·4 | *De Novo* | Williams Beuren syndrome (WBS) region. | #194050/ Li et al. 2016 |
| IT_FLO_076 | Deletion | 7q11.23 | 72726378 | 74139531 | 1·4 | Unknown | Williams Beuren syndrome (WBS) region. | #194050/ Li et al. 2016 |
| IT_FLO_120 | Deletion | 7q11.22-q21.3 | 69492424 | 93170601 | 23.7 | *De Novo* | Contains Williams Beuren syndrome (WBS) region. | #194050 |
| IT_FLO_010 | Deletion | 14q12 | 28069160 | 30673249 | 2.6 | De Novo | 14q12 FOXG1 deletion syndrome | *164874/Kortüm et al.2011 |
| IT_FLO_053 | Deletion | 14q12 | 28556165 | 31442017 | 2.9 | Unknown | 14q12 FOXG1 deletion syndrome | *164874/Kortüm et al.2011 |
| IT_FLO_156 | Deletion | 14q13.1 | 32084785 | 33740209 | 1.7 | *De Novo* | 14q11-q22 deletion syndrome | #613457 |
| UK_L_132 | Duplication | 15q11.1q13.3 | 20205266 | 32719882 | 12.5 | Unknown | 15q11.2 duplication syndrome, distal | #608636 |
| IT_FLO_139 | Duplication | 15q.11.1-q13.2 | 20102541 | 30322079 | 10.2 | *De Novo* | 15q11.2 duplication syndrome, distal | #608636 |
| BE_LEU_233 | Duplication | 15q11.2 | 22299329 | 28726651 | 6.4 | Unknown | 15q11.2 duplication syndrome, distal | #608636 |
| BE_LEU_008 | Duplication | 16p11.2 | 29678746 | 30013045 | 3.3 | *De Novo* | 16p11.2 duplication syndrome | #614671 |
| IT_FLO_018 | Duplication | 16p11.2 | 29652999 | 30197290 | 5.4 | *De Novo* | 16p11.2 duplication syndrome | #614671 |
| IT_FLO_148 | Duplication | 16p11.2 | 29673954 | 30198600 | 0.5 | Inherited (Paternal) | 16p11.2 duplication syndrome | #614671 |
| UK_L_164 | Duplication | 16p11.2 | 29785758 | 30093458 | 0.3 | Unknown | 16p11.2 duplication syndrome | #614671 |
| IT_FLO_113 | Deletion | 17p11.2 | 16763408 | 20133702 | 3.4 | Unknown | Smith Magenis deletion syndrome | #182290 |
| IT_FLO_047 | Duplication | 17p11.2 | 16664000 | 20234000 | 3.5 | *De Novo* | Potocki-Lupski duplication syndrome | #610883 |
| IT_FLO_091 | Duplication | 17p11.2 | 16795275 | 20192408 | 3.4 | *De Novo* | Potocki-Lupski duplication syndrome | #610883 |
| BE_LEU_085 | Deletion | 17p13.3 | 2540435 | 2826082 | 0.3 | Unknown | Miller- Dieker Lissencephaly deletion syndrome | #247200 |
| BE_LEU_107 | Deletion | 17p13.3 | 2403985 | 2554321 | 0.2 | *De Novo* | Miller- Dieker Lissencephaly deletion syndrome | #247200 |
| IT_FLO_005 | Deletion | 17p13.3 | 2157250 | 2537250 | 0.4 | *De Novo* | Miller- Dieker Lissencephaly deletion syndrome | #247200 |
| UK_L_193 | deletion | 17q11.2 | 29473264 | 29812256 | 0.3 | Unknown | 17q11.2 deletion syndrome | #162200 |
| IT_FLO_050 | Deletion | 17q21.3 | 43706217 | 44210223 | 0.5 | *De Novo* | Koolen-De Vries deletion syndrome | #610443 |
| IT_FLO_123 | Deletion | 17q21.31 | 43717503 | 44277147 | 0.6 | *De Novo* | Koolen-De Vries deletion syndrome | #610443 |
| BE_ANT_097 | Deletion | 18q22.2 | 68426681 | 70626821 | 2.2 | *De Novo* | 18q deletion syndrome | #601808 |
| BE_VIB_020 | Deletion | 18q22.2-q22.3 | 68424195 | 70619647 | 2.2 | *De Novo* | 18q deletion syndrome | #601808 |
| IT_FLO_004 | Deletion | 21q22.13-q22.3 | 38755130 | 43310931 | 4.5 | *De Novo* | Keppen-Lubinsky syndrome region. | #614098 |
| IT_FLO_015 | Deletion | 22q13.31-q13.33 | 47663336 | 51218134 | 3.5 | Unknown | Phelan-McDermid deletion syndrome | #606232 |

*e. Table S.4b*: Autosomal CNVs deemed pathogenic by size (> 3 Mb or <3 and > 1 Mb and *de novo*)

| **Individual** | **CNV Type** | **Chr region** | **Start** | **Stop** | **Size (Mb)** | **Inheritance** | **Epilepsy phenotype** | **Other phenotype** | **Neuroimaging** | **Decipher (associated phenotype)** | **DGV** |
| --- | --- | --- | --- | --- | --- | --- | --- | --- | --- | --- | --- |
| BE_ANT_004 | Deletion | 1q42.13 | 227098588 | 243181599 | 16.1 | Unknown | Generalized of unknown origin | ID; microcephaly; dysmorphisms; persistent ductus arteriosus;kidney defects; dysplastic hip | CC agenesis, Arnold Chiari malformation | Similar CNV (facial abnormalities kidney, CC agenesis, hip dysplasia, ID) | Not present |
| BE_LEU_099 | Duplication | 2q31.1 | 172679936 | 180914725 | 8.2 | Unknown | Nos epilepsy | ID, Syndactyly | Periventricular leukomalacia | Pathogenic in Decipher (ID, seizures) | Not present |
| BE_LEU_144 | Duplication | 4q35.1 | 184560009 | 190966890 | 6.4 | Unknown | Focal of unknown origin | ID, Hirschprung disease, cryptorchidism, facial dysmorphisms | Negative | Pathogenic in Decipher (ID and seizures) | Not present |
| IT_FLO_061 | Deletion | 4q35.1 | 184431851 | 190883851 | 6.5 | *De Novo* | Generalized of unknown origin | ID, facial dysmorphisms | Negative | Partial overlap with two pathogenic CNVs:4:182074317-190791091;4:182074317-190807379 with ID | Not present |
| UK_L_228 | Duplication | 5p15.33p13.3 | 91650 | 33063190 | 33 | Unknown | Myoclonic epilepsy of infancy | ID, Torticollis, pyloric stenosis, plagiocephaly | Negative | This region contains a CNV defined as pathogenic in decipher (no phenotype data ) | Not present |
| BE_ANT_139 | Deletion | 5p15.32 | 5743661 | 9716549 | 4 | Inherited (P) | Nos epilepsy | ID, autism, amblyopia | Negative | Not present | Not present |
|  |  |  |  |  |  |  |  |  |  |  |  |
| BE_LEU_189 | Deletion | 5p15.33-p32 | 2682974 | 5982763 | 3.3 | Inherited (P) | Nos epilepsy | Autism, anorexia | Ventriculo-peritoneal shunt (hydrocephaly), bilateral hippocampal malrotation | Not present | Not present |
| IT_FLO_116 | Deletion | 5q14.3-q15 | 88860018 | 93148083 | 4.3 | Unknown | Focal of unknown origin | ID, facial dysmorphisms | Negative | Not present | Not present |
| PO_W_034 | Deletion | 5q14.3-q15 | 89654817 | 94557076 | 4.9 | *De Novo* | Nos epilepsy | ID, facial dysmorphisms | NA | Pathogenic in Decipher (deeply set eye, frontal bossing, ID, peripheral dysmyelination, protruding ear, seizures, spasticity) | Not present |
| PO_W_028 | Deletion | 5q35.1-q35.3 | 171909167 | 177949176 | 6 | Unknown | Nos epilepsy | ID, facial dysmorphisms | NA | Partial overlap with a pathogenic deletion 5:174961216-178038146); contains a smaller likely pathogenic CNV (abnormality of cardiovascular system morphology and macrocephaly) | Not present |
| IT_FLO_132 | Deletion | 6q12-q14.1 | 69291497 | 76786292 | 7.5 | *De Novo* | Generalized of unknown origin | Dysgraphia, dyslexia | Negative | Not present | Not present |
| BE_ANT_045 | Deletion | 8q22.2 | 99225337 | 104490503 | 5.3 | Unknown | Generalized of unknown origin | ID, bilateral ptosis, small stature slow growth, microcephaly | NA | Not present | Not present |
| IT_FLO_067 | Deletion | 8q22.2-q22.3 | 100973253 | 105261783 | 4.3 | *De Novo* | Generalized of unknown origin | ID, hypotonia, facial dysmorphisms | Negative | Includes a pathogenic deletion in Decipher (absence seizures) | Not present |
| PO_W_029 | Deletion | 10p14-p12.31 | 10555403 | 22173062 | 11.6 | *De Novo* | Focal of structural origin | ID, facial dysmorphisms | Focal cortical dysplasia | Not present | Not present |
| BE_LEU_144 | Deletion | 10p15.3 | 116829 | 6739705 | 6.6 | Unknown | Focal of unknown origin, DR | Hirschprung disease, cryptorchidism, facial dysmorphisms | NA | Pathogenic in Decipher (ID, seizures) | Not present |
| BE_LEU_242 | Deletion | 10p15.3 | 116829 | 3822976 | 3.7 | Unknown | Nos epilepsy | global developmental delay, quadriplegia, abnormal facial shape | Pachygyria | Pathogenic in Decipher (abnormal facial shape, language deficit, epileptic spasms, global DD, IDs, severe pachygyria, seizures, tetraplegia) | Not present |
| BE_LEU_039 | Duplication | 10q11.22 | 47553107 | 51804955 | 4.3 | Unknown | Nos epilepsy | ID, small for gestational age, facial dysmorphisms | NA | This region maps inside a larger region reported as pathogenic (no phenotype) | Not present |
| BE_LEU_113 | Duplication | 10q11.22 | 46405262 | 51804955 | 5.4 | *De Novo* | Nos epilepsy | ID | NA | Pathogenic in Decipher (ID, seizures) | Not present |
| BE_LEU_193 | Deletion | 10q22.3 | 81641918 | 88940418 | 7.3 | Unknown | Nos epilepsy | mild developmental delay, hypotonia | NA | Partial overlapping pathogenic CNVs in Decipher (DD, seizures) | Not present |
| PO_W_030 | Deletion | 10q26.2-q26.3 | 129741462 | 135524747 | 5.8 | Inherited (M) | Focal of unknown origin | ID, facial dysmorphisms | NA | Partial overlapping pathogenic CNVs in Decipher (ID, DD) | Not present |
|  |  |  |  |  |  |  |  |  |  | Included in the 10q26 deletion syndrome |  |
| IT_FLO_070 | Deletion | 11q24.2-25 | 126210701 | 134927255 | 8.7 | Unknown | Focal of unknown origin | facial dysmorphisms, somatic hypoplasia | NA | Not present | Not present |
| BE_LEU_102 | Duplication | 12q24.31 | 125250201 | 128510145 | 3.3 | Inherited (M) | Focal of structural origin | ID, autism, pubertas precox (central) | Periventricular leukomalacia, hypoplastic optic nerve | Partial overlapping with a pathogenic CNVs in Decipher (different phenotypes) | Not present |
| BE_VIB_015 | Deletion | 13q13.1q13.3 | 33957317 | 36828237 | 2.9 | *De Novo* | Epileptic encephalopathy | ID, hypotonia, ataxia, behavioral problems | Negative | Partial overlapping with a pathogenic CNVs in Decipher (different phenotypes) | Not present |
|  |  |  |  |  |  |  |  |  |  |  |  |
|  |  |  |  |  |  |  |  |  |  |  |  |
| IT_FLO_025 | Deletion | 13q33.1-q34 | 107115182 | 115169878 | 8.1 | Unknown | Nos epilepsy | ID, behavioural disturbances, microcephaly, facial dysmorphisms, language disorder | Negative | Partial overlap with pathogenic CNVs in decipher (ID, DD, microcephaly) | Not present |
| IT_FLO_139 | Duplication | 15q13.2-q13.3 | 31014508 | 32914081 | 1.9 | *De Novo* | Nos epilepsy | ID, hypotonia | Negative | Pathogenic in Decipher (ID, DD, seizure, autism, language delay) | Not present |
| IT_FLO_065 | Deletion | 15q21.3-22.1 | 57283554 | 59255080 | 2 | *De Novo* | Generalized of unknown origin , FS | Craniostenosis | Negative | Not present | Not present |
| BE_LEU_026 | Deletion | 16q22.1 | 70052138 | 73541249 | 3.5 | Unknown | Nos epilepsy | Bilateral talipes equinovarus, facial dysmorphisms, mitral valve defect, decreased central vision, urinary incontinence, hearing impairment | NA | Not present | Not present |
| IT_FLO_122 | Duplication | 17q11.1-q11.2 | 25403246 | 26596219 | 1.2 | *De Novo* | Focal of unknown origin | ID | Negative | Not present | Not present |
| IT_FLO_074 | Duplication | 19q13.33-q13.43 | 49739516 | 59092570 | 9.4 | *De Novo* | Nos epilepsy | Bilateral deafness, facial dysmorphisms | PNH | Not present | Not present |
| BE_ANT_122 | Duplication | 19q13.11 | 32764189 | 33921542 | 1.2 | *De Novo* | Nos epilepsy of structural origin | ID, mild dysmorphic features | Polymicrogyria | Not present | Not present |
| US_073 | Duplication | 20p13-20p12.1 | 60001 | 14536200 | 14.5 | Unknown | Focal epilepsy of unknown origin | ID. Ataxia, spasticity, kyphoscoliosis, aortic valve deficiency | Enlarged lateral ventricles with pronunciation of occipital horns (colpocephaly) | Not present | Not present |
| UK_L_253 | Duplication | 20p12.3-20p12.1 | 8302410 | 14665080 | 6.4 | Unknown | Generalized of unknown origin | ID, hypotonia, facial dysmorphisms | Thickening and enhancement of the dura grey matter signal changes | Not present | Not present |
| IT_FLO_133 | Duplication | 20q11.2 | 29888277 | 37763670 | 7.9 | *De Novo* | Generalized of unknown origin | Language disorder, ADHD, syndactyly, | Negative | Not present | Not present |

*Legend table S.4b:* ADHD: attention deficit hyperactivity disorder; FS: febrile seizures; CC: corpus callosum; DD: developmental delay ; DR: drug-resistant; ID: intellectual disability; M, maternal; NA: not available; Nos: not otherwise specified; P: paternal; PNH: periventricular nodular heterotopia; CSWS: continuous spike and waves during sleep.

*g. Table S.5*: Patients with CNVs including a) *HNRNPU* or b) *RORB*

a. Patients with CNVs including *HNRNPU*

| **ID** | **Gender** | **YOB** | **Seizure onset** | **Seizure types** | **EEG** | **AEDs** | **Epilepsy outcome** | **Epilepsy type/syndromes** | **Psychomotor development** | **Head size** | **Other features** | **MRI** | **Epilepsy genes** | **CNV Type** |
| --- | --- | --- | --- | --- | --- | --- | --- | --- | --- | --- | --- | --- | --- | --- |
| IT_FLO_041 | F | 2008 | 1 m | focal epilepsy with secondary generalization | slow background, multifocal paroxysmal abnormalities increased during sleep | PB, VPA, CBZ, LEV, RFN, LTG, CLB, CLN, ZNS | Drug resistant epilepsy with daily seizures | Infantile onset drug resistant epilepsy | Severe ID | Congenital microcephaly (< 4 SD) | Stereotypic movements, scolyosis, hypotonia, facial dysmorphisms: hypertelorism, long philtrum, thin upper lip, micrognathia, dysmorphic large auricles | CC agenesia, holoprosencephaly | *HNRNPU, AKT3* | Deletion |
| BE_LEU_127 | F | 2011 | neonatal | neonatal onset seizures (possibly intra-uterine?) | bursts of posterior sharp activity, hight voltage slow delta activity | LEV | Patient deceased (respiratory insuff.) | Infantile onset unclassified epilepsy | Hypotonic at birth | 36.5 cm at birth (normal) | Dysmorphic features: downslanting of eyes, hypertelorism, straight eyebrows, long fingers; cardial: large aorta ascendens and aortic arch, open ductus Botalli, left/right shunt; pulmonary hypoplasia; malrotation of kidneys | Small widening of lateral ventricles and cavum vergae, polymicrogyria | *HNRNPU, AKT3* | Dupication. |
| PO_W_031 | F | 2008 | 7 m | Complex partial seizures; clusters | Focal spikes, spike-waves complexes in anterior area | VPA,LEV | Seizures partially controlled by therapy, (febrile seizures ongoing) | Infantile onset drug resistant epilepsy | ID, autism | Microcephaly (<3 SD) | Hypotonia,camptocormia, poor growth, facial dysmorphisms: upslanding palpebral fissures, epicanthal folds, synophrys, broad face, low forehead, flat nasal bridge, broad thick lips, dysmorphic teeth, micrognathia hypertelorism, long philtrum, thin upper lip, micrognathia, dysmorphic large auricles, exotropia | Frontal lobe atrophy and CC hypoplasia | *HNRNPU, AKT3* | Deletion |
| IT_FLO_062 | F | 1992 | 6 m | Generalized | Generalized spike and polyspike-wave discharges; 2-3 Hz spike-wave discharges | PB, VPA, CBZ, ESM, LTG | Absences, monthly frequence | GGE | Moderate ID | Acquired microcephaly (-2 SD) | Scolyosis,hyposomia, growth hormone deficiency, deafness, joint hyperlaxity, facial dysmorphisms | CC hypoplasia, ventricles asymmetry | *HNRNPU* | Deletion |
| BE_LEU_009 | M | 1990 | 4 y | FS, tonic, myoclonic | Slow background, multifocal paroxysmal activity increased during sleep | TPM, VPA, CLB, LTG | Controlled on LTG and VPA | Lennox Gastaut Syndrome | Severe ID | Normal | Flexion contractures of the 4 limbs, scoliosis, left spastic hemiparesis (wheelchair bound), gastro-esophagus reflux, bilatearl corneal opacity | Delayed myelination, atrophic septum pellucidum, aqueduct stenosis, hydrocephaly | *HNRNPU* | Deletion |

b. Patients with CNVs including *RORB*

| **ID** | **Gender** | **YOB** | **Seizure onset** | **Seizure types** | **EEG** | **AEDs** | **Epilepsy outcome** | **Epilepsy type/syndromes** | **Psychomotor development** | **Other features** | **MRI** | **Epilepsy genes** | **CNVs Type** |
| --- | --- | --- | --- | --- | --- | --- | --- | --- | --- | --- | --- | --- | --- |
| PO_W_019 | M | 1998 | 4 y | Absences with eyelids myoclonia, GTCS | Generalized spikes/polyspikes-wave complexes. multifocal spikes; CSWS. IPS+ ( IV type) | VPA, ETS | Absence seizures with eyelids myoclonia | Generalized photosentive epilepsy (Jeavons Syndrome) | Severe ID, Autism | None | Negative | *RORB* | Deletion |
| BE_LEU_244 | F | 2002 | 3 y | Generalized, atonic, atypical absences + eyelid myoclonia | Epileptic activity with 2 Hz spike-waves complexes | VPA, CLN | Seizure-free on VPA and CLN | Generalized epilepsy of unknown origin | Global developmental delay | Episodic ataxia | Small non-specific white matter lesions over right parietal hemisphere | *RORB* | Deletion |
| US_267 | M | 1993 | 4-5 y | Absences, generalized tonic clonic seizures | Rare bifrontal spike waves | VPA | Good response to VPA, long period of sz freedom after start VPA | Generalized of unknown origin | Severe ID | Pyramidal sign, tremor, neurogenic bladder, psychotic episodes, severe macrocytic anemia, cold agglutinin disease, bilateral femuropatellar arthrosis, facial dysmorphisms | NA | RORB | Partial Deletion |

*Legend tables S.5a and S.5b:* CBZ, carbamazepine; CC, corpus callosum;CLB, clobazam; CLN, clonazepam; CSWS: continuous spike-waves during slow sleep; ESM, ethosuximide; FU, follow-up; GGE, genetic generalized epilepsy; GTCS: generalized tonic clonic seizures; HC, head circumference; ID, intellectual disability; IPS: intermittent photic stimulation, LEV, levetiracetam; LTG, lamotrigine; m: months; NA, not available; PB, phenobarbital; RFN, rufinamide; VPA, valproic acid; y:year; YOB, year of birth.

*h. Table S6* Literature data about a) *HNRNPU*  or b) *RORB*

*a) literature about HNRNPU*

| **Mutation** | **Age of onset** | **EEG** | **Epilepsy type/syndromes** | **Epilepsy outcome** | **Psychomot development** | **Other clinical features** | **MRI** | **Ref.** |
| --- | --- | --- | --- | --- | --- | --- | --- | --- |
| p.Thr582_Gln589del | 16 months | Burst of sharp and slow wave activity during sleep | Lennox-Gastaut syndrome | DR epilepsy | ID, language absence | Autistic and behavioral disorder | PVNH | ^64^ ^1,65^ |
| p.Tyr805* | 2y | Generalysed Spike and polyspike wave, diffuse slowing | Lennox-Gastaut syndrome |  | ID severe | NA | / | ^1^ |
| p.Gln171* | 12 months | NA | GTC epilepsy | Rare seizures | ID | Dysmorphisms, autistic spectrum disorder, aortic dilatation | Broad Sylvian fissures, enlarged sub-arachnoid spaces, white matter abnormalities in the periventricular and the right frontal sub-cortical  region | ^65,66^ |
| 5' splice site alteration | 7years old | NA | Nos Epilepsy | NA | ID | Hypertension, bone abnormalities | / | ^67^ |
| p.E236Tfs*6 (de novo) | 8 months | Epileptiform activity | Nos Epilepsy | NA | ID | Hyperlaxity, hypotonia | Delayed myelination | ^68^ |
| pV604 fs *24 (de novo) | NA | NA | Epileptic encephalopathy | NA | NA | NA | / | ^69^ |
| p.Val6Ilefs*4 | 2.5 months | NA | Tonic, clonic/ nos epilpsy | NA | ID, severe | Global hypotonia | Enlarged lateral ventricles, complete CC | ^70^ |
| p.Asn767Glufs*66 (mosaic) | NA | NA | NA | NA | Moderate/severe ID | Axial hypotonia, spastic diplegia | Dilated ventricles (presumed aqueduct stenosis), small splenium CC | ^70^ |
| p.Glu624Argfs*24 | 8 months | NA | GTCS, atonic/nos epilepsy | NA | ID severe | Hypotonia, hyperlaxity of joints | Normal | ^70^ |
| p.? C.2425-3C>A | 7 months | NA | Absences, GTCS/nos epilepsy | NA | Severe ID, autism | NA | Normal (CT scan performed before the age of 4) | ^70^ |
| p.Gln561Serfs*45 | 4 months | NA | Atypical absences, GTCS/ nos epilepsy | NA | Severe ID | None | ThiN CC | ^70^ |
| p.Gln561* mosaic | 24 months | NA | GTCS, absences/nos epilepsy | NA | Moderate ID | Hypotonia | Normal | ^70^ |
| p.? C.692-1G>A | 1-2 months | NA | Nos epilepsy | NA | Moderate-severe ID | NA | NA | ^70^ |
| p.Gly218Alafs*118 | 7 months | NA | West syndrome | Rare seizures | ID | None | Malrotated left hippocampus | ^71^ |
| p.Arg572* | 14 months | NA | Lennox-Gastaut syndrome | DR epilepsy | ID | None | Absence of left olfactory sulcus and left olfactory bulb | ^71^ |
| p.Trp363* | 9 months | NA | Complex partial, febrile/nos epilepsy | DR epilepsy | ID | Autism | Decreased T2 shortening in the anterior temporal lobes | ^71^ |
| p.Pro757Argfs*7 | 2 years old | NA | Atypical absences, febrile/nos epilepsy | Rare seizures | ID | Stereotypies, constipation, mild hypertrichosis, hipermobility, unilateral single palmar crease | Normal | ^71^ |
| p.(Lys543*) | 5 years old | NA | febrile | NA | moderate ID | Dysmorphisms, dry skin | Normal | ^72^ |
| p.(Glu140Lys) | NA | NA | NA | NA | Moderate ID, Tourette syndrome | Transposition of great vessels, dysmorphisms, | Small periventricular areas with high T2 signal | ^72^ |
| Splice donor site alteration | 1 year old | NA | NA | NA | Moderate ID, Autism | Dysmorphisms, spinal lordosis | Normal | ^72^ |
| p.(Val8Glufs*4) | 5 years old | NA | NA | Only one seizure | Severe ID | Dysmorphisms | Normal | ^72^ |
| p.(Ile476Profs*7) | <1 year old | NA | NA | NA | Severe ID | Dysmorphisms | Normal | ^72^ |
| p.(Trp320*) | 8 months | NA | NA | NA | Severe ID | Dysmorphisms | Normal | ^72^ |
| p.(Leu555Argfs*51) | 18 months | NA | NA | NA | Moderate ID | NA | Non progressive T2 and Flair hyperintensities in white matter bilaterally | ^72^ |
| p.(Arg324Gly) | 14 months | Hypsarrhythmia | Myoclonic epilepsy | Rare seizures | Severe ID | Dysmorphisms, scoliosis, cardial abnormalities | Abnormal corpus callosum, cerebral atrophy, glial liesions, atrophy of cerebellar vermis | ^65^ |
| p.(Gln273*) | NA | NA | Febrile seizures | NA | Severe ID | Dysmorphisms, cardial abnormalities | NA | ^65^ |
| Duplication, estimated: chr1: 245,025,709- 245,133,797 | Neonatal | Normal | Focal myoclonies | NA | Severe ID | Microcephaly, dysmorphisms, hypotonia, cardial abnormalities, autism, arthrogryposis, | Cystic lesion ; Dandy Walker malformation | ^65^ |
| p.(Ser378Pro) | 10 months | NA | Temporal epilepsy evolving into West syndrome evolved in Lennox-Gastaut-like epilepsy with multiple daily absences, temporal tonic and infrequent atonic seizures, sometimes fever-related | DR epilepsy | severe ID | Hypotonia, autism, cortical visual impairment, nystagmus | Wide ventricles, retarded frontal myelinisation, glandula pinealis cyst | ^65^ |
| p.(Gln175*) | 11 months | NA | Febrile TCS , absences, later occurring without fever | NA | Severe ID | Dysmorphisms, strabismus | Normal | ^65^ |

*b) literature about RORB*

| **Individual (gender)** | **Chr region/RORB mutation** | **Age of onset** | **Epilepsy** | **EEG** | **Epilepsy type/syndromes** | **Epilepsy outcome** | **Psychomot development** | **Ref.** |
| --- | --- | --- | --- | --- | --- | --- | --- | --- |
| Family 1 | p.Arg66* (familial, 4 patients) | 3-9 y | Absence seizured, GTCs, photosesitivity | Generalyzed polyspikes 3Hz spike and waves | GGE (absences, eyelid and neck and shoulder myoclonia) | Seizures controlled by drugs | Mild ID | ^73^ |
| Patient AG1 (F) | p.Leu73Pro (de novo) | 3y | Febrile and afebrile GTCs , positive family history for febrile seizures | NA | Febrile and afebrile GTCs | Seizures controlled by VPA | Moderate-severe ID with regression, autism | ^73^ |
| Patient ROI (M) | p.Thr417del (de novo) | 4 m | Tonic seizures, clonic-atonic seizures , atypical absences | NA | Tonic, clonic-atonic seizures , atypical absences | Drug resistant epilepsy | Severe ID | ^73^ |
| Case9A1117(M) | 9q21 del including RORB and additional 47 genes | 6 y | Bilateral clonic febrile seizures, tonic-clonic, absence seizures | Typical 3Hz spike and wave | Absence seizures, oral or limb automatisms | DR epilepsy | ID | ^73^ |
| Case GE 0705 (F) | 9q21 del including RORB ex 1-5 | 4.5 y | Absence seizures, GTCs |  | Absence seizures, eyelid myoclonia, | DR | ID, speech impairment | ^73^ |
| Case DK8393 (F) | 9q21 t(9;19) (q21;q12) | No seizures | No seizures |  |  |  | Moderate ID, autism | ^73^ |
| Case EC-CAE300 | 9q21 del including RORB exon 1 + promoter | 2-3 y | GTCs, absence seizures, |  |  | Seizures controlled by drugs | Aggressive behavior | ^74^ |
| Case | p.L73P, de novo |  | NA | NA | NA | NA | Autism | ^69^ |

*Legend tables S.6a and S.6b*: CC: corpus callosum DR: drug -resistant; F:female; GGE: genetic generalized epilepsy ; GTCs: generalized tonic clonic seizures; ID: intellectual disability; m: months; M:male; NA: not available; nos: not otherwise specified; PVNH: periventricular nodular heterotopia; y: years

Table 7S: CNVs having a different classification before/after application of the workflow

| **Individual** | **CNV type** | **Chr region** | **Start** | **Stop** | **Size (Mb)** | **Inheritance** | | **Original classification** | | **Classification after application of the workflow** | **Cause of the different classification** |
| --- | --- | --- | --- | --- | --- | --- | --- | --- | --- | --- | --- |
|  |  |  |  |  |  |  |  |  |  |  | **(by applying our workflow)** |
| IT_FLO_036 | Duplication | 4q21.22-q21.23 | 84035965 | 84813544 | 0.8 | *De novo* | | Unknown significance | | Possibly pathogenic | Includes brain- expressed gene *MRPS18C* and *de novo*,. Also reported from another group^75^ |
| US_184 | Duplication | 3q28 | 191886383 | 192432844 | 0.5 | *De novo* | | Unknown significance | | Possibly pathogenic | Includes brain expressed gene *FGF12* and *de novo*. Recently associated with epilepsy ^76^ |
| IT_FLO_127 | Deletion | 5q23.2 | 122481284 | 122987185 | 0.5 | *De novo* | | Unknown significance | | Possibly pathogenic | Includes brain expressed gene *CSNK1G3* and *de novo* |
| US_175 | Deletion | 16p13.2 | 8368145 | 8860296 | 0.5 | Inherited (M) | | Unknown significance | | Possibly pathogenic | Includes gene ABAT (AR), considered an epilepsy gene according to Table S.2: suppl material. |
| BE_LEU_009 | Duplication | 1q43 | 239842929 | 240356854 | 0.5 | *De novo* | | Pathogenic | | Possibly pathogenic | Includes two AR brain expressed genes *FMN2, CHRM3* and de novo |
| PO_W_030 | Duplication | 20q13.33 | 61925286 | 62724437 | 0.8 | Inherited (M) | | Pathogenic | | Possibly pathogenic | Includes epilepsy genes *CHRNA4, KCNQ2, EEF1A2*. Inherited, phenotype unknown, |
| BE_ANT_122 | Duplication | 19q13.11 | 32764189 | 33921542 | 1.2 | *De novo* | | Unknown significance | | CNVs deemed pathogenic by size | Size <3 and > 1 Mb, *de novo*, includes many brain expressed genes |
| US_185 | Deletion | 15q11.2 | 22766227 | 23110280 | 0.3 | Inherited (P) | | Unknown significance | | Pathogenic Recurrent CNVs | Overlapping with pathogenic CNVs reported by ^1,74^ |
| BE_ANT_139 | Deletion | 5p15.32 | 5743661 | 9716549 | 4 | | Inherited (P) | | Unknown significance | CNVs deemed pathogenic by size | Size >3Mb, includes many brain expressed genes |
| UK_L_193 | Deletion | 17q11.2 | 29473264 | 29812256 | 0.3 | | Unknown | | Unknown significance | Pathogenic CNVs related to a genetic OMIM syndrome | Overlapping with pathogenic CNVs reported. Includes an epilepsy gene *NF1,* unknown inheritance |

*i. Figure S.1***:** Autosomal CNVs per samples across all samples included in the study (N=1097).

*Legend figure S.1*: Number of autosomal CNV calls per sample for the study cohort.

*j. Figure S.2:* MAQ electropherogram


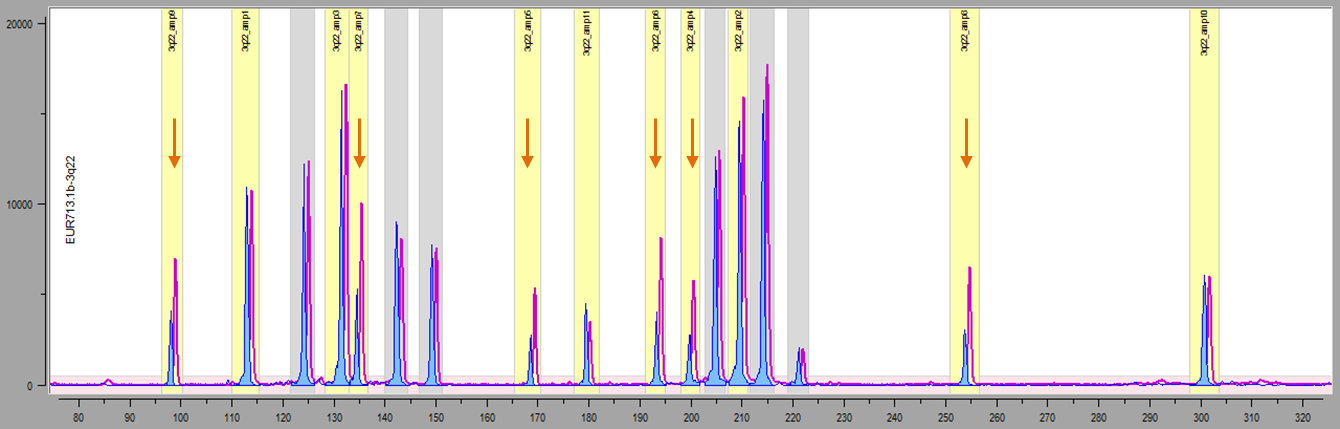


*Legend figure S.2*: MAQ 3q22 electropherogram. Arrows indicate peak areas where the patient sample shows half of the amplification compared to the reference samples. Blue peaks indicate areas of amplification from the patient sample; purple peaks are from the reference sample. Yellow bins correspond to the target amplicons in the CNV region, grey bins refer to control amplicons located at randomly selected genomic positions.

### *j. Figure S.3: Composite forest plot of meta-analysis of pathogenic CNV yield in patients with intellectual disability (a); psychiatric/neurological disorders (b); epilepsy without EE (c).*

| a) | 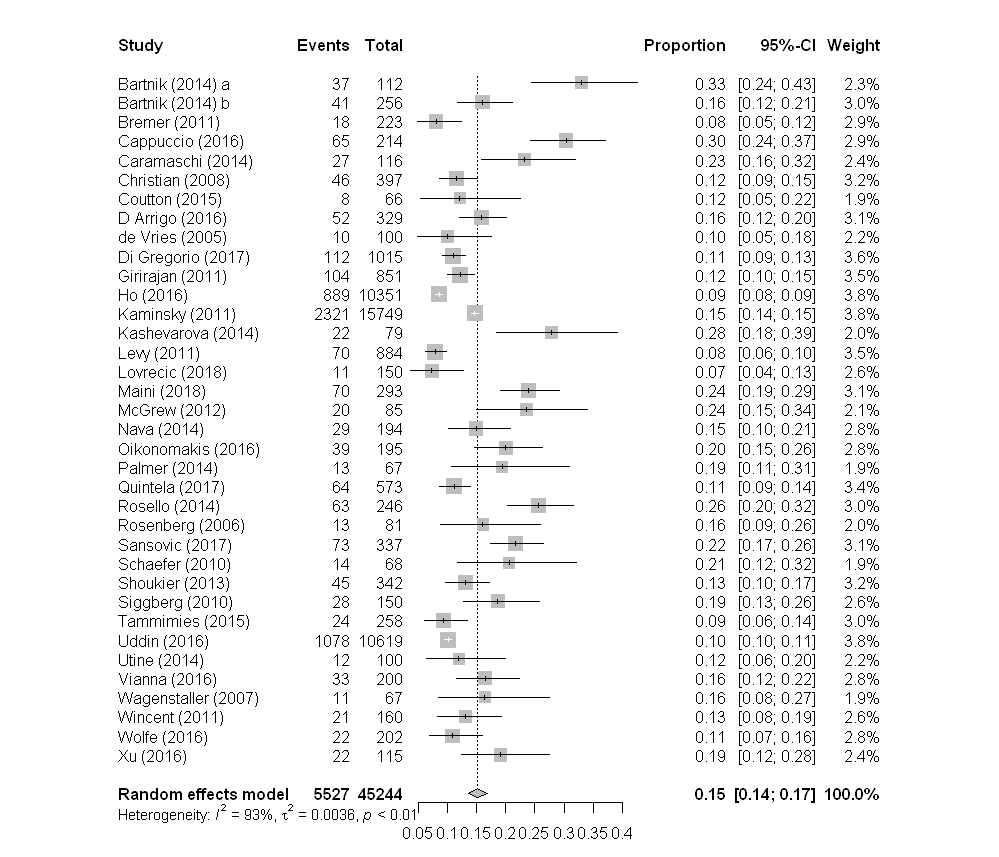 |
| --- | --- |
| b) | 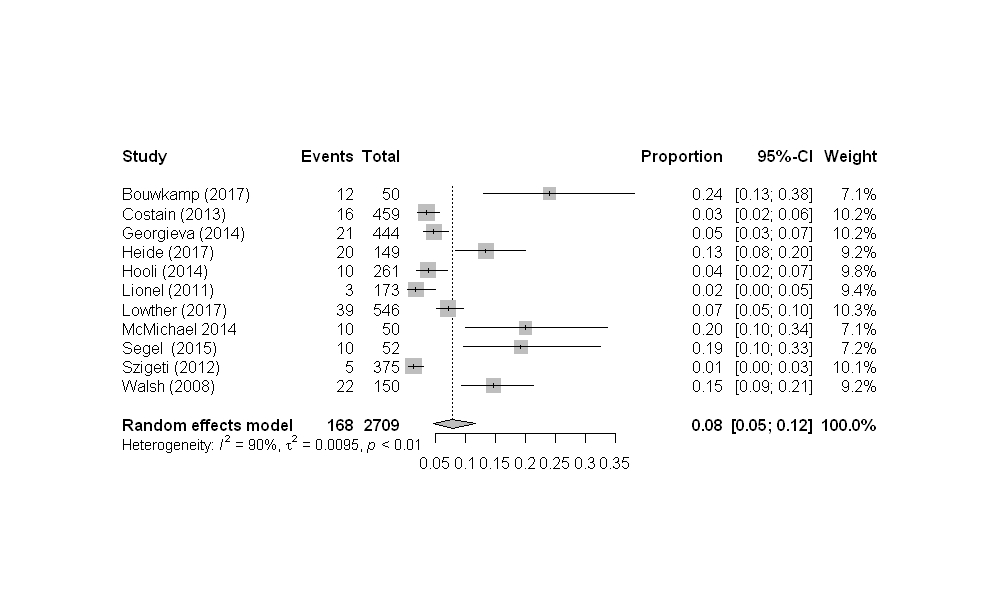 |

| c) | 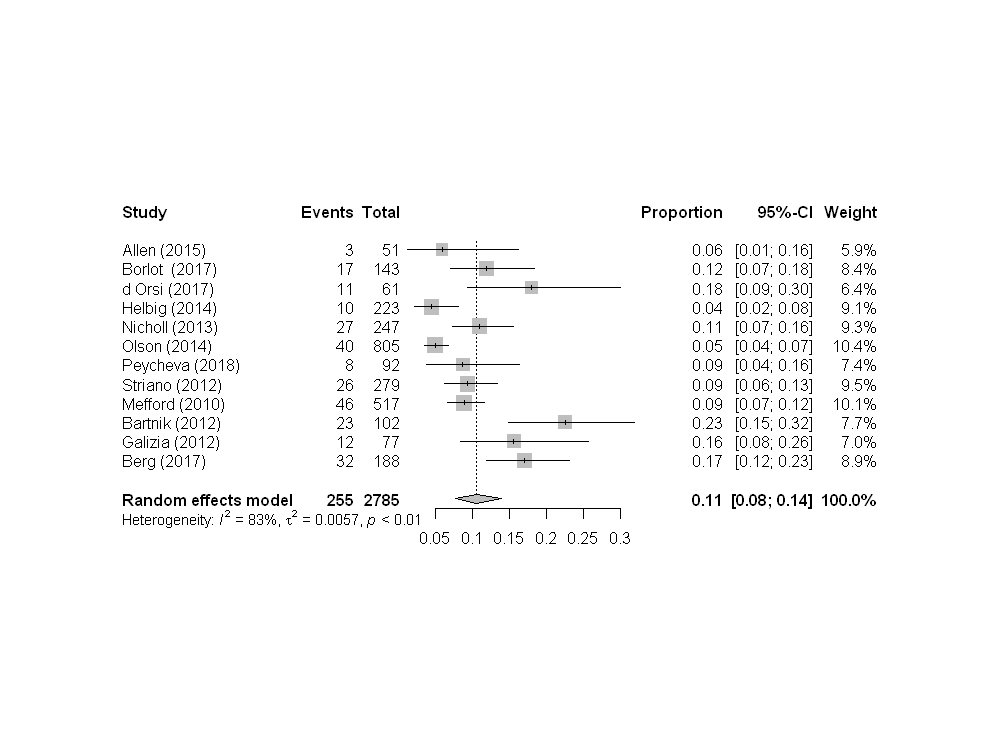 |
| --- | --- |

L*egend figure S.3*:

The squares represent the study-specific proportions. The size of each square represents the weight of each study in the [meta-analysis](https://www.ncbi.nlm.nih.gov/books/n/cer40/appendixes.app4/def-item/appendixes.app4.gl1-d20/). Horizontal lines through each square represent 95% [confidence intervals](https://www.ncbi.nlm.nih.gov/books/n/cer40/appendixes.app4/def-item/appendixes.app4.gl1-d8/). The diamonds represent the pooled proportions.

**3. Supplemental References:**

1. Carvill GL, Mefford HC. Microdeletion syndromes. *Curr Opin Genet Dev* 2013; **23**(3): 232-9.

2. Zarrei M, MacDonald JR, Merico D, Scherer SW. A copy number variation map of the human genome. *Nat Rev Genet* 2015; **16**(3): 172-83.

3. Krumm N, O'Roak BJ, Shendure J, Eichler EE. A de novo convergence of autism genetics and molecular neuroscience. *Trends Neurosci* 2014; **37**(2): 95-105.

4. Pinto D, Delaby E, Merico D, et al. Convergence of genes and cellular pathways dysregulated in autism spectrum disorders. *Am J Hum Genet* 2014; **94**(5): 677-94.

5. Mefford HC, Muhle H, Ostertag P, et al. Genome-wide copy number variation in epilepsy: novel susceptibility loci in idiopathic generalized and focal epilepsies. *PLoS Genet* 2010; **6**(5): e1000962.

6. Striano P, Coppola A, Paravidino R, et al. Clinical Significance of Rare Copy Number Variations in Epilepsy: A Case-Control Survey Using Microarray-Based Comparative Genomic Hybridization. *Arch Neurol* 2011.

7. Helbig I, Swinkels ME, Aten E, et al. Structural genomic variation in childhood epilepsies with complex phenotypes. *Eur J Hum Genet* 2014; **22**(7): 896-901.

8. Olson H, Shen Y, Avallone J, et al. Copy number variation plays an important role in clinical epilepsy. *Ann Neurol* 2014; **75**(6): 943-58.

9. Allen NM, Conroy J, Shahwan A, et al. Chromosomal microarray in unexplained severe early onset epilepsy - A single centre cohort. *Eur J Paediatr Neurol* 2015; **19**(4): 390-4.

10. Kearney HM, Thorland EC, Brown KK, Quintero-Rivera F, South ST, Working Group of the American College of Medical Genetics Laboratory Quality Assurance C. American College of Medical Genetics standards and guidelines for interpretation and reporting of postnatal constitutional copy number variants. *Genet Med* 2011; **13**(7): 680-5.

11. Bartnik M, Szczepanik E, Derwinska K, et al. Application of array comparative genomic hybridization in 102 patients with epilepsy and additional neurodevelopmental disorders. *Am J Med Genet B Neuropsychiatr Genet* 2012; **159B**(7): 760-71.

12. Bartnik M, Nowakowska B, Derwinska K, et al. Application of array comparative genomic hybridization in 256 patients with developmental delay or intellectual disability. *J Appl Genet* 2014; **55**(1): 125-44.

13. Bartnik M, Wisniowiecka-Kowalnik B, Nowakowska B, et al. The usefulness of array comparative genomic hybridization in clinical diagnostics of intellectual disability in children. *Dev Period Med* 2014; **18**(3): 307-17.

14. Berg AT, Coryell J, Saneto RP, et al. Early-Life Epilepsies and the Emerging Role of Genetic Testing. *JAMA Pediatr* 2017; **171**(9): 863-71.

15. Borlot F, Regan BM, Bassett AS, Stavropoulos DJ, Andrade DM. Prevalence of Pathogenic Copy Number Variation in Adults With Pediatric-Onset Epilepsy and Intellectual Disability. *JAMA Neurol* 2017; **74**(11): 1301-11.

16. Bouwkamp CG, Kievit AJA, Markx S, et al. Copy Number Variation in Syndromic Forms of Psychiatric Illness: The Emerging Value of Clinical Genetic Testing in Psychiatry. *Am J Psychiatry* 2017; **174**(11): 1036-50.

17. Bremer A, Giacobini M, Eriksson M, et al. Copy number variation characteristics in subpopulations of patients with autism spectrum disorders. *Am J Med Genet B Neuropsychiatr Genet* 2011; **156**(2): 115-24.

18. Cappuccio G, Vitiello F, Casertano A, et al. New insights in the interpretation of array-CGH: autism spectrum disorder and positive family history for intellectual disability predict the detection of pathogenic variants. *Ital J Pediatr* 2016; **42**: 39.

19. Caramaschi E, Stanghellini I, Magini P, et al. Predictive diagnostic value for the clinical features accompanying intellectual disability in children with pathogenic copy number variations: a multivariate analysis. *Ital J Pediatr* 2014; **40**: 39.

20. Christian SL, Brune CW, Sudi J, et al. Novel submicroscopic chromosomal abnormalities detected in autism spectrum disorder. *Biol Psychiatry* 2008; **63**(12): 1111-7.

21. Costain G, Lionel AC, Merico D, et al. Pathogenic rare copy number variants in community-based schizophrenia suggest a potential role for clinical microarrays. *Hum Mol Genet* 2013; **22**(22): 4485-501.

22. Coutton C, Dieterich K, Satre V, et al. Array-CGH in children with mild intellectual disability: a population-based study. *Eur J Pediatr* 2015; **174**(1): 75-83.

23. D'Arrigo S, Gavazzi F, Alfei E, et al. The Diagnostic Yield of Array Comparative Genomic Hybridization Is High Regardless of Severity of Intellectual Disability/Developmental Delay in Children. *J Child Neurol* 2016; **31**(6): 691-9.

24. de Vries BB, Pfundt R, Leisink M, et al. Diagnostic genome profiling in mental retardation. *Am J Hum Genet* 2005; **77**(4): 606-16.

25. Di Gregorio E, Riberi E, Belligni EF, et al. Copy number variants analysis in a cohort of isolated and syndromic developmental delay/intellectual disability reveals novel genomic disorders, position effects and candidate disease genes. *Clin Genet* 2017; **92**(4): 415-22.

26. d'Orsi G, Martino T, Palumbo O, et al. The epilepsy phenotype in adult patients with intellectual disability and pathogenic copy number variants. *Seizure* 2017; **53**: 86-93.

27. Galizia EC, Srikantha M, Palmer R, et al. Array comparative genomic hybridization: results from an adult population with drug-resistant epilepsy and co-morbidities. *Eur J Med Genet* 2012; **55**(5): 342-8.

28. Georgieva L, Rees E, Moran JL, et al. De novo CNVs in bipolar affective disorder and schizophrenia. *Hum Mol Genet* 2014; **23**(24): 6677-83.

29. Girirajan S, Brkanac Z, Coe BP, et al. Relative burden of large CNVs on a range of neurodevelopmental phenotypes. *PLoS Genet* 2011; **7**(11): e1002334.

30. Heide S, Keren B, Billette de Villemeur T, et al. Copy Number Variations Found in Patients with a Corpus Callosum Abnormality and Intellectual Disability. *J Pediatr* 2017; **185**: 160-6 e1.

31. Ho KS, Wassman ER, Baxter AL, et al. Chromosomal Microarray Analysis of Consecutive Individuals with Autism Spectrum Disorders Using an Ultra-High Resolution Chromosomal Microarray Optimized for Neurodevelopmental Disorders. *Int J Mol Sci* 2016; **17**(12).

32. Hooli BV, Kovacs-Vajna ZM, Mullin K, et al. Rare autosomal copy number variations in early-onset familial Alzheimer's disease. *Mol Psychiatry* 2014; **19**(6): 676-81.

33. Kaminsky EB, Kaul V, Paschall J, et al. An evidence-based approach to establish the functional and clinical significance of copy number variants in intellectual and developmental disabilities. *Genet Med* 2011; **13**(9): 777-84.

34. Kashevarova AA, Nazarenko LP, Skryabin NA, et al. Array CGH analysis of a cohort of Russian patients with intellectual disability. *Gene* 2014; **536**(1): 145-50.

35. Levy D, Ronemus M, Yamrom B, et al. Rare de novo and transmitted copy-number variation in autistic spectrum disorders. *Neuron* 2011; **70**(5): 886-97.

36. Lionel AC, Crosbie J, Barbosa N, et al. Rare copy number variation discovery and cross-disorder comparisons identify risk genes for ADHD. *Sci Transl Med* 2011; **3**(95): 95ra75.

37. Lovrecic L, Rajar P, Volk M, et al. Diagnostic efficacy and new variants in isolated and complex autism spectrum disorder using molecular karyotyping. *J Appl Genet* 2018; **59**(2): 179-85.

38. Lowther C, Merico D, Costain G, et al. Impact of IQ on the diagnostic yield of chromosomal microarray in a community sample of adults with schizophrenia. *Genome Med* 2017; **9**(1): 105.

39. Maini I, Ivanovski I, Djuric O, et al. Prematurity, ventricular septal defect and dysmorphisms are independent predictors of pathogenic copy number variants: a retrospective study on array-CGH results and phenotypical features of 293 children with neurodevelopmental disorders and/or multiple congenital anomalies. *Ital J Pediatr* 2018; **44**(1): 34.

40. McGrew SG, Peters BR, Crittendon JA, Veenstra-Vanderweele J. Diagnostic yield of chromosomal microarray analysis in an autism primary care practice: which guidelines to implement? *J Autism Dev Disord* 2012; **42**(8): 1582-91.

41. McMichael G, Girirajan S, Moreno-De-Luca A, et al. Rare copy number variation in cerebral palsy. *Eur J Hum Genet* 2014; **22**(1): 40-5.

42. Nava C, Keren B, Mignot C, et al. Prospective diagnostic analysis of copy number variants using SNP microarrays in individuals with autism spectrum disorders. *Eur J Hum Genet* 2014; **22**(1): 71-8.

43. Nicholl J, Waters W, Suwalski S, et al. Epilepsy with cognitive deficit and autism spectrum disorders: Prospective diagnosis by array CGH. *Am J Med Genet B Neuropsychiatr Genet* 2013; **162**(1): 24-35.

44. Oikonomakis V, Kosma K, Mitrakos A, et al. Recurrent copy number variations as risk factors for autism spectrum disorders: analysis of the clinical implications. *Clin Genet* 2016; **89**(6): 708-18.

45. Palmer E, Speirs H, Taylor PJ, et al. Changing interpretation of chromosomal microarray over time in a community cohort with intellectual disability. *Am J Med Genet A* 2014; **164A**(2): 377-85.

46. Peycheva V, Kamenarova K, Ivanova N, et al. Chromosomal microarray analysis of Bulgarian patients with epilepsy and intellectual disability. *Gene* 2018; **667**: 45-55.

47. Quintela I, Eiris J, Gomez-Lado C, et al. Copy number variation analysis of patients with intellectual disability from North-West Spain. *Gene* 2017; **626**: 189-99.

48. Rosello M, Martinez F, Monfort S, Mayo S, Oltra S, Orellana C. Phenotype profiling of patients with intellectual disability and copy number variations. *Eur J Paediatr Neurol* 2014; **18**(5): 558-66.

49. Sansovic I, Ivankov AM, Bobinec A, Kero M, Barisic I. Chromosomal microarray in clinical diagnosis: a study of 337 patients with congenital anomalies and developmental delays or intellectual disability. *Croat Med J* 2017; **58**(3): 231-8.

50. Schaefer GB, Starr L, Pickering D, Skar G, Dehaai K, Sanger WG. Array comparative genomic hybridization findings in a cohort referred for an autism evaluation. *J Child Neurol* 2010; **25**(12): 1498-503.

51. Segel R, Ben-Pazi H, Zeligson S, et al. Copy number variations in cryptogenic cerebral palsy. *Neurology* 2015; **84**(16): 1660-8.

52. Shoukier M, Klein N, Auber B, et al. Array CGH in patients with developmental delay or intellectual disability: are there phenotypic clues to pathogenic copy number variants? *Clin Genet* 2013; **83**(1): 53-65.

53. Siggberg L, Ala-Mello S, Jaakkola E, et al. Array CGH in molecular diagnosis of mental retardation - A study of 150 Finnish patients. *Am J Med Genet A* 2010; **152A**(6): 1398-410.

54. Szigeti K, Lal D, Li Y, et al. Genome-wide scan for copy number variation association with age at onset of Alzheimer's disease. *J Alzheimers Dis* 2013; **33**(2): 517-23.

55. Tammimies K, Marshall CR, Walker S, et al. Molecular Diagnostic Yield of Chromosomal Microarray Analysis and Whole-Exome Sequencing in Children With Autism Spectrum Disorder. *JAMA* 2015; **314**(9): 895-903.

56. Uddin M, Pellecchia G, Thiruvahindrapuram B, et al. Indexing Effects of Copy Number Variation on Genes Involved in Developmental Delay. *Sci Rep* 2016; **6**: 28663.

57. Utine GE, Haliloglu G, Volkan-Salanci B, et al. Etiological yield of SNP microarrays in idiopathic intellectual disability. *Eur J Paediatr Neurol* 2014; **18**(3): 327-37.

58. Vianna GS, Medeiros PF, Alves AF, Silva TO, Jehee FS. Array-CGH analysis in patients with intellectual disability and/or congenital malformations in Brazil. *Genet Mol Res* 2016; **15**(1).

59. Wagenstaller J, Spranger S, Lorenz-Depiereux B, et al. Copy-number variations measured by single-nucleotide-polymorphism oligonucleotide arrays in patients with mental retardation. *Am J Hum Genet* 2007; **81**(4): 768-79.

60. Walsh T, McClellan JM, McCarthy SE, et al. Rare structural variants disrupt multiple genes in neurodevelopmental pathways in schizophrenia. *Science* 2008; **320**(5875): 539-43.

61. Wincent J, Anderlid BM, Lagerberg M, Nordenskjold M, Schoumans J. High-resolution molecular karyotyping in patients with developmental delay and/or multiple congenital anomalies in a clinical setting. *Clin Genet* 2011; **79**(2): 147-57.

62. Wolfe K, Strydom A, Morrogh D, et al. Chromosomal microarray testing in adults with intellectual disability presenting with comorbid psychiatric disorders. *Eur J Hum Genet* 2016; **25**(1): 66-72.

63. Xu Q, Goldstein J, Wang P, et al. Chromosomal microarray analysis in clinical evaluation of neurodevelopmental disorders-reporting a novel deletion of SETDB1 and illustration of counseling challenge. *Pediatr Res* 2016; **80**(3): 371-81.

64. Epi KC, Epilepsy Phenome/Genome P, Allen AS, et al. De novo mutations in epileptic encephalopathies. *Nature* 2013; **501**(7466): 217-21.

65. Bramswig NC, Ludecke HJ, Hamdan FF, et al. Heterozygous HNRNPU variants cause early onset epilepsy and severe intellectual disability. *Hum Genet* 2017; **136**(7): 821-34.

66. Hamdan FF, Srour M, Capo-Chichi JM, et al. De novo mutations in moderate or severe intellectual disability. *PLoS Genet* 2014; **10**(10): e1004772.

67. Need AC, Shashi V, Hitomi Y, et al. Clinical application of exome sequencing in undiagnosed genetic conditions. *J Med Genet* 2012; **49**(6): 353-61.

68. de Kovel CG, Brilstra EH, van Kempen MJ, et al. Targeted sequencing of 351 candidate genes for epileptic encephalopathy in a large cohort of patients. *Mol Genet Genomic Med* 2016; **4**(5): 568-80.

69. Farwell KD, Shahmirzadi L, El-Khechen D, et al. Enhanced utility of family-centered diagnostic exome sequencing with inheritance model-based analysis: results from 500 unselected families with undiagnosed genetic conditions. *Genet Med* 2015; **17**(7): 578-86.

70. Depienne C, Nava C, Keren B, et al. Genetic and phenotypic dissection of 1q43q44 microdeletion syndrome and neurodevelopmental phenotypes associated with mutations in ZBTB18 and HNRNPU. *Hum Genet* 2017; **136**(4): 463-79.

71. Leduc MS, Chao HT, Qu C, et al. Clinical and molecular characterization of de novo loss of function variants in HNRNPU. *Am J Med Genet A* 2017; **173**(10): 2680-9.

72. Yates TM, Vasudevan PC, Chandler KE, et al. De novo mutations in HNRNPU result in a neurodevelopmental syndrome. *Am J Med Genet A* 2017; **173**(11): 3003-12.

73. Rudolf G, Lesca G, Mehrjouy MM, et al. Loss of function of the retinoid-related nuclear receptor (RORB) gene and epilepsy. *Eur J Hum Genet* 2016.

74. Lal D, Ruppert AK, Trucks H, et al. Burden analysis of rare microdeletions suggests a strong impact of neurodevelopmental genes in genetic generalised epilepsies. *PLoS Genet* 2015; **11**(5): e1005226.

75. Ottaviani V, Bartocci A, Pantaleo M, et al. MYOCLONIC ASTATIC EPILEPSY IN A PATIENT WITH A DE NOVO 4q21.22q21.23 MICRODUPLICATION. *Genet Couns* 2015; **26**(3): 327-32.

76. Shi RM, Kobayashi T, Kikuchi A, et al. Phenytoin-responsive epileptic encephalopathy with a tandem duplication involving FGF12. *Neurol Genet* 2017; **3**(1): e133.
